# 80 years of extracellular vesicles: from discovery to clinical translation

**DOI:** 10.20517/evcna.2025.161

**Published:** 2026-02-10

**Authors:** Qiang Li, Yinan Ding, Yejiao Shi, Chong Qiu, Lei Lei, Shenglong Li, Zheng Zhu, Judun Zheng, Cheng Qin, Kaiyuan Wang, Cheng Jiang, Ziyi Han, Lingyan Yang, Lang Zhang, Ping Li, Lingjun Tong, Duan Wang, Hong Xu, Bingyang Dai, Yangyang Du, Kaiyang Wang, Zhijin Fan, Wei Wang, Keying Guo, Yu Huang, Xia Wang, Bingdong Sui, Liang Wen, Feixiong Chen, Dechao Feng, Xiang Qin, Wenjun Mao, Hongxing Liu, Chen Liu, Zhaoting Li, Yongfei Wang, Ru Huang, Rong Lu, Yulin Zhang, Ying Tian, Xiaolong Miao, Yuan Yin, Jun Zhang, Zhizeng Wang, Teng Ma, Haifeng Dong, Daixu Wei, Zhengyang Yang, Xiaohong Yang, Xiaoyu Cheng, Wojciech Chrzanowski, Zhigang Chang, Xudong Zhang, William C. Cho, Yang Luo, Weiliang Xia, Zhaohui Huang

**Affiliations:** ^1^Department of Critical Care Medicine, Beijing Hospital, National Center of Gerontology, Institute of Geriatric Medicine, Chinese Academy of Medical Sciences, Beijing 100730, China.; ^2^Zhejiang Cancer Hospital, Hangzhou Institute of Medicine (HIM), Chinese Academy of Sciences, Hangzhou 310022, Zhejiang, China.; ^3^Institute of Translational Medicine, Shanghai University, Shanghai 200444, China.; ^4^Artemisinin Research Center, and Institute of Chinese Materia Medica, China Academy of Chinese Medical Sciences, Beijing 100700, China.; ^5^Ministry of Education, Provincial Key Laboratory of Biotechnology, College of Life Sciences, Northwest University, Xi’an 710069, Shaanxi, China.; ^6^Cancer Hospital of China Medical University, Liaoning Cancer Hospital & Institute, Shenyang 110042, Liaoning, China.; ^7^Department of Urology, Xijing Hospital, Fourth Military Medical University, Xi’an 710032, Shaanxi, China.; ^8^Dermatology Hospital, Southern Medical University, Guangzhou 510091, Guangdong, China.; ^9^Peking Union Medical College Hospital, Chinese Academy of Medical Sciences and Peking Union Medical College, Beijing 100730, China.; ^10^Wuya College of Innovation, Shenyang Pharmaceutical University, Shenyang 110016, Liaoning, China.; ^11^School of Medicine, The Chinese University of Hong Kong, Shenzhen 518172, Guangdong, China.; ^12^Zhongda Hospital, Medical School of Southeast University, Nanjing 210009, Jiangsu, China.; ^13^Guangzhou National Laboratory, Guangzhou 510005, Guangdong, China.; ^14^Institute of Rheumatology and Immunology, Affiliated Hospital of North Sichuan Medical College, Nanchong 637000, Sichuan, China.; ^15^Cheeloo College of Medicine, Shandong University, Qingdao 266237, Shandong, China.; ^16^Medical Science and Technology Innovation Center, Shandong First Medical University & Shandong Academy of Medical Sciences, Jinan 250117, Shandong, China.; ^17^Department of Orthopedic Surgery and Orthopedic Research Institution, West China Hospital, Sichuan University, Chengdu 610041. Sichuan, China.; ^18^Department of Biomedical Engineering, Faculty of Engineering, The Hong Kong Polytechnic University, Hong Kong SAR, China.; ^19^Changchun Institute of Applied Chemistry, Chinese Academy of Sciences, Changchun 130022, Jilin, China.; ^20^Department of Orthopedic Surgery, Shanghai Sixth People’s Hospital Affiliated to Shanghai Jiao Tong University School of Medicine, Shanghai 200233, China.; ^21^Institute for Engineering Medicine, Kunming Medical University, Kunming 650500, Yunnan, China.; ^22^Department of Biotechnology and Food Engineering, Guangdong Technology Israel Institute of Technology, Shantou 515063, Guangdong, China.; ^23^Xinqiao Hospital, Third Military Medical University, Chongqing 400037, China.; ^24^The First Affiliated Hospital, Guangzhou Medical University, Guangzhou 510120, Guangdong, China.; ^25^School of Stomatology, The Fourth Military Medical University, Xi’an 710032, Shaanxi, China.; ^26^The First Affiliated Hospital, Zhejiang University School of Medicine, Hangzhou 310003, Zhejiang, China.; ^27^Faculty of Biochemistry and Molecular Medicine, University of Oulu, Oulu 90014, Finland.; ^28^Division of Surgery & Interventional Science, University College London, London WC1E 6BT, UK.; ^29^School of Life Science and Technology, University of Electronic Science and Technology of China, Chengdu 610054, Sichuan, China.; ^30^Wuxi People’s Hospital, Wuxi Medical Center, Nanjing Medical University, Wuxi 214023, Jiangsu, China.; ^31^Tianjin Medical University Cancer Institute and Hospital, National Clinical Research Center for Cancer, Tianjin 300060, China.; ^32^School of Biomedical Engineering, Hainan University, Sanya 572025, Hainan, China.; ^33^Marine College, Shandong University, Weihai 264209, Shandong, China.; ^34^Cheeloo College of Medicine, Shandong University, Jinan 250012, Shandong, China.; ^35^Organ Transplantation Center, The Affiliated Hospital of Qingdao University, Qingdao 266003, Shandong, China.; ^36^Wuxi Cancer Institute, Affiliated Hospital of Jiangnan University, Wuxi 214062, Jiangsu, China.; ^37^Department of Orthopedics, The First Affiliated Hospital of Chongqing Medical University, Chongqing 400016, China.; ^38^Department of Laboratory Medicine, Chongqing Center for Clinical Laboratory, Chongqing Academy of Medical Sciences, Chongqing General Hospital, School of Medicine, Chongqing University, Chongqing 401147, China.; ^39^Department of Molecular Pharmaceutics, University of Utah, Salt Lake City, UT 84112, USA.; ^40^School of Biomedical Engineering, Shenzhen University Medical School, Shenzhen University, Shenzhen 518060, Guangdong, China.; ^41^Clinical Medical College and Affiliated Hospital of Chengdu University, Chengdu University, Chengdu 610081, Sichuan, China.; ^42^Beijing friendship hospital, Capital medical university, Beijing 100050, China.; ^43^Chongqing Institute of Green and Intelligent Technology, Chinese Academy of Sciences, Chongqing 400714, China.; ^44^College of Optical Science and Engineering, Zhejiang University, Hangzhou 310052, Zhejiang, China.; ^45^Faculty of Medicine and Health, The University of Sydney, Sydney 2006, Australia.; ^46^School of Medicine, Sun Yat-sen University, Shenzhen 518107, Guangdong, China.; ^47^Department of Clinical Oncology, Queen Elizabeth Hospital, Hong Kong SAR, China.; ^48^Chongqing General Hospital, School of Medicine, Chongqing University, Chongqing 401147, China.; ^49^Center for Aging and Cancer Research, Global Institute of Future Technology, Shanghai Jiao Tong University, Shanghai 200240, China.; ^#^These authors contributed equally to this work.

**Keywords:** Extracellular vesicles, exosomes, microvesicles, apoptotic bodies, intercellular communication, drug delivery, liquid biopsy

## Abstract

Extracellular vesicles (EVs) are heterogeneous, lipid bilayer-enclosed vesicles secreted by cells. Research on EVs dates back to the 1940s, and the term “exosomes” - a major subtype of EVs - was coined in 1981 to describe small membrane vesicles shed from cells. However, it is only in the past two decades that research in this area has expanded rapidly. By transferring functional biomolecules, EVs play a pivotal role in intercellular communication and regulate a wide range of cellular functions under both physiological and pathological conditions. Owing to their high biocompatibility, capacity to protect encapsulated cargo from degradation, and ability to cross biological barriers, EVs also show great promise as biomarkers and drug-delivery systems. Following the first, albeit unintentional, isolation of EVs in 1946, the 80th anniversary of EV research is now approaching. In this review, we trace the history of EV research and summarize key advances in the field. We also discuss current challenges and future prospects in this rapidly evolving area.

## INTRODUCTION

Extracellular vesicles (EVs) are heterogeneous populations of membrane-enclosed vesicles secreted by cells. According to the International Society for Extracellular Vesicles (ISEV), EVs are broadly categorized by size into large EVs (> 200 nm) and small EVs (sEVs, < 200 nm)^[1]^, while others classify EVs into three main subtypes, including exosomes, microvesicles (MVs), and apoptotic bodies, based on their biogenesis pathways^[2,3]^. Exosomes, typically 30-150 nm in diameter, largely overlap in size with sEVs and are generated by cells via the fusion of multivesicular bodies (MVBs) with the plasma membrane. As a key subpopulation of EVs, exosomes play a crucial role in intercellular communication. Given their pivotal roles in various physiological and pathological processes, exosomes have attracted considerable research interest across multiple research areas, particularly in cancer biology, immunology, and regenerative medicine^[2-5]^.

The study of EVs dates back to 1946, when Chargaff *et al*. observed a clotting factor resembling the thromboplastic protein, a finding now regarded as one of the earliest observations related to EVs. By the 1970s, several studies had reported the existence of EVs both *in vitro* and *in vivo*^[6]^. The term “exosome” was first used by Fox *et al*. in 1970 to describe DNA fragments transferred between Drosophila or Neurospora cells^[7,8]^. In 1981, Trams *et al*. referred to exfoliated small membrane vesicles as exosomes^[9]^. Direct evidence of exosomes emerged in 1983, when they were observed in sheep reticulocytes during their maturation process^[10,11]^. In 1987, Johnstone *et al*. formally designated these small vesicles as exosomes^[12]^. These early discoveries laid the foundation for subsequent EV research. Initially considered mere cellular waste products, exosomes gained recognition in the late 1990s and early 2000s, when studies revealed their critical function in intercellular communication through transporting bioactive molecules. This revelation transformed exosomes from cellular debris to active participants in physiological and pathological processes^[13-15]^. Now, EVs are seen as promising candidates for biomarkers, therapeutic delivery systems, and therapeutic targets in a variety of medical fields^[16-18]^. From these early explorations to current cutting-edge applications in disease diagnosis and treatment, the field of EV research has undergone a remarkable evolution over the past 50 years.

In this review, we provide a comprehensive overview of the 50-year path of EV research, tracing its progression from basic discovery to clinical applications. We first review their discovery, biogenesis, composition, and unique biological properties. Subsequently, we summarize existing methods for EVs isolation and characterization. Then, we discuss the biological roles and mechanisms of EVs, emphasizing their growing importance in biomedical research. Finally, we address the challenges in translating EV-based research into clinical applications and explore potential future directions in this rapidly developing field.

## HISTORY OF EVs

### Discovery and naming of exosomes and EVs

Exosomes are small, spherical, or cup-shaped membranous vesicles generated by cells through the process of endocytosis followed by secretion. They are released into the extracellular space after MVBs are integrated with the plasma membrane, which distinguishes them from other types of EVs^[19]^. Exosomes have a lipid bilayer membrane structure and carry various bioactive molecules, including proteins, lipids, RNA, and DNA fragments, which reflects the biological status and functions of their parent cells.

Research on exosomes over the past four decades has evolved from initial descriptive observations to in-depth functional and mechanistic investigations. The field was formally inaugurated in 1987 when Johnstone *et al*. discovered small vesicles with a membrane structure in the supernatant of sheep red blood cell cultures and termed them exosomes^[12]^. Since then, exosomes have been increasingly recognized as vital mediators of intercellular communication, capable of transporting various signaling molecules such as cytosolic proteins, signal transduction proteins, metabolic enzymes, heat shock proteins (HSP), and tetraspanins^[20,21]^.

Exosomes are secreted by almost all cell types and widely distributed in various body fluids, including serum, plasma, saliva, urine, cerebrospinal fluid, and breast milk. Their widespread distribution and ease of manipulation make exosomes a hot topic in biomedical research, including disease diagnosis, drug delivery, disease therapy, and biomimetic technologies^[22]^.

### Definitions and standards of exosomes and EVs

It is important to note that early EV studies often failed to clearly distinguish between exosomes, MVs, ectosomes, and other EV subtypes. Since the release of the Minimal Information for Studies of Extracellular Vesicles (MISEV) 2018 guidelines^[1]^, the terms “exosome” and “small extracellular vesicles (sEVs)” have sometimes been used interchangeably in cell biology and biomedical research. However, they represent distinct entities with different definitions and contextual implications^[4]^. Exosomes are specifically defined as vesicles derived from MVBs and released through the fusion of these organelles with the plasma membrane^[4,23]^. MVBs are organelles containing multiple small vesicles that are formed by budding from the membrane within the MVB. The generation of exosomes is thus a multistep process involving endosomal membrane budding and fusion mechanisms. As a result, exosomes are typically rich in certain protein markers, such as tetraspanins CD9, CD63, and CD81, which are commonly used for exosome identification^[22]^.

In contrast, the term “sEVs” refers to vesicles smaller than 200 nm, a classification based primarily on physical size and methodological considerations. Consequently, this category includes not only exosomes but also other small vesicles generated through different biogenetic pathways. The definition of sEVs emphasizes physical properties and offers a more inclusive classification, acknowledging practical challenges in rigorously distinguishing exosome subgroups from other vesicular structures (e.g., MVs) in experimental settings. To improve reproducibility and consistency across studies, ISEV recommends using operational terms such as “sEV” unless the endosomal origin of vesicles is unequivocally demonstrated. Current standardization efforts face challenges due to overlapping marker profiles and functional attributes among sEV subpopulations, as well as cell type-dependent variations in vesicle characteristics. Therefore, researchers are encouraged to provide clear descriptions of isolation methods and identification markers tailored to their study aims. Using both “exosome” and “sEV” appropriately - depending on the biological and experimental context - can help convey precise scientific meaning and facilitate the integration of research findings^[1,18,24]^.

### Milestone events in EV research

The history of EV research can be traced back to 1946, when Erwin Chargaff and Randolph West hypothesized the existence of small blood cell fragments in blood samples subjected to high-speed centrifugation^[25]^. In 1967, Walter Halperin and William Jensen observed ultrastructural changes in cultured carrot plant cells by electron microscopy, highlighting the distinct features of meristematic cells^[26]^. In the same year, Peter Wolf captured the first electron microscopy images of platelet-derived EVs, providing crucial visual evidence for the field^[27]^. In 1974, Christian de Duve was awarded the Nobel Prize for the discovery of lysosomes, laying an important foundation for vesicle transport research^[28]^. In 1981, Trams *et al*. first used the term “exosome” to describe small vesicles isolated from the culture media of normal and tumor cells^[9]^. In 1983, two groups independently discovered small vesicles in sheep reticulocytes nearly simultaneously^[10,11]^, which were later confirmed to be exosomes by Johnstone *et al*. in 1987^[12]^.

The 1990s marked a shift from morphological description to functional exploration. Exosomes were initially thought to function primarily in waste disposal but were later implicated in immune regulation. A key breakthrough came in 1996 when Raposo *et al*. reported that B lymphocyte-derived exosomes could present antigens^[29]^, a finding subsequently extended to dendritic cells (DCs) by Zitvogel *et al*. in 1998^[30]^. In 1999, the first EV-related clinical trial was posted on ClinicalTrials.gov^[31]^.

The early 21st century witnessed a deeper understanding on exosome biogenesis and their role as intercellular messengers. In 2001, research revealed that intestinal epithelial cells released exosome-like vesicles capable of modulating immune responses^[32]^. In 2006, Ratajczak *et al*. demonstrated for the first time that MVs derived from embryonic stem cells carried not only proteins but also messenger RNA (mRNA), further expanding their role in intercellular communication^[33]^. In the subsequent year, Valadi *et al*. confirmed the existence of both mRNA and microRNA (miRNA) molecules in exosomes, suggesting their capacity for genetic exchange between cells and opening avenues for RNA-based therapies^[14]^. In 2009, Aslam *et al*. demonstrated the significant therapeutic potential of exosomes from bone marrow stromal cells in the treatment of bronchopulmonary dysplasia^[34]^.

In the 2010s, the potential of exosomes as diagnostic biomarkers and therapeutic delivery vehicles gained increasing attention, leading to in-depth research into their involvement in various diseases, including cancer, neurological disorders, and cardiovascular diseases (CVDs). In 2013, Rothman, Schekman, and Südhof were awarded the Nobel Prize for their research into vesicle transport mechanisms^[35]^. In 2016, McKiernan *et al*. developed a urine exosome gene expression assay (ExoDx) to predict high-grade prostate cancer^[36]^. In 2017, Kamerkar *et al*. constructed engineered exosomes to deliver small interfering RNA (siRNA) targeting oncogenic Kras^G12D^ (Kirsten rat sarcoma virus) in pancreatic cancer^[37]^. Concurrently, exosome isolation and characterization technologies, including ultracentrifugation, size-exclusion chromatography (SEC), and advanced imaging techniques, have been significantly improved^[38]^. To standardize information in the rapidly expanding field, ISEV released its first MISEV guidelines in 2014^[39]^, which were updated in 2018^[1]^. In 2015, Huan *et al*. demonstrated that exosomes derived from acute myeloid leukemia could inhibit hematopoietic stem cells in the bone marrow microenvironment^[40]^. In the same year, Raghu Kalluri identified circulating glypican-1 (GPC1)^+^ exosomes as a promising screening tool for pancreatic cancer^[41]^, while Li *et al*. reported that circular RNAs (circRNAs) are enriched and stable in exosomes, providing the first evidence of exosomal circRNAs as novel biomarkers^[42]^. In 2018, Cai *et al*. discovered that plant exosome-like EVs could deliver small RNAs to fungal pathogens for cross-kingdom gene silencing^[43]^. In the same year, Li *et al*., Lai *et al*. and Yu *et al*. developed the first exosomal RNA database, exoRBase, a repository for circRNA, long non-coding RNA (lncRNA) and mRNA in human biofluids^[44-46]^, thereby promoting basic and translational research on EV-associated RNAs (EV RNAs) in human diseases.

**Figure 1:**
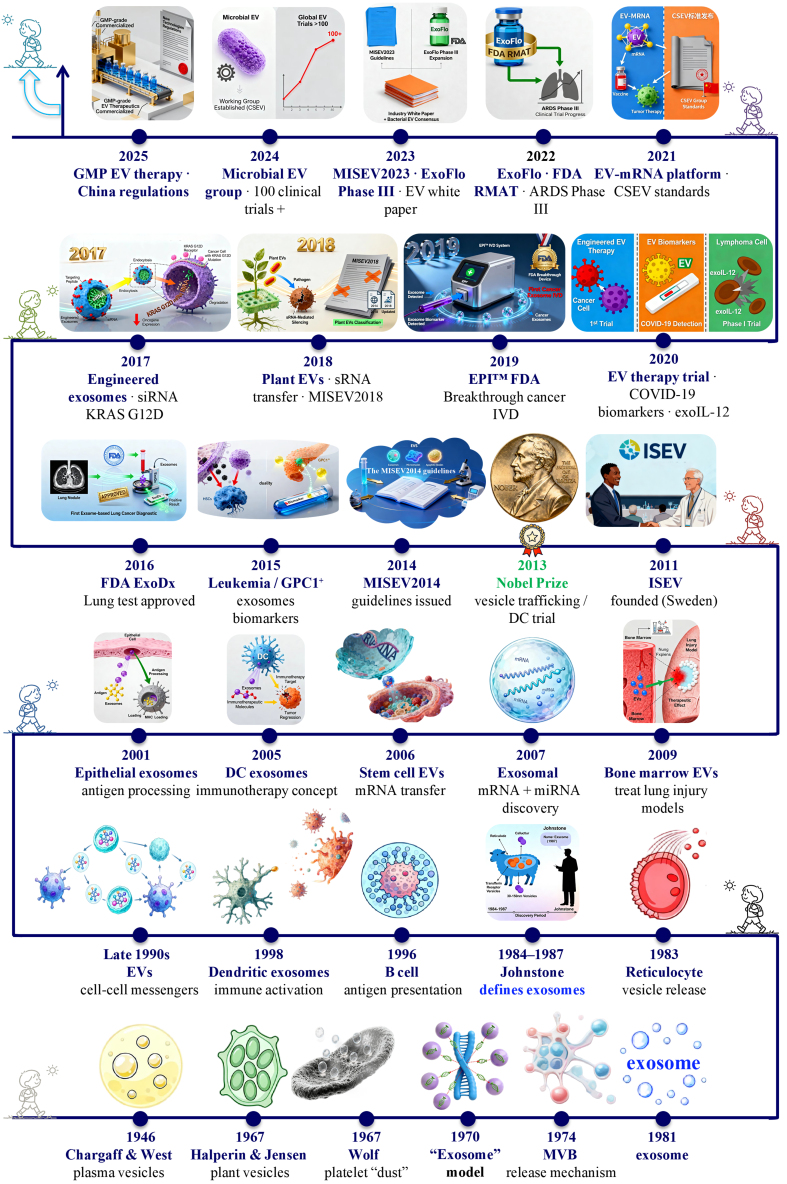
Milestones in the discovery, conceptual evolution, and clinical translation of EVs from 1946 to 2025. This timeline charts eight decades of progress, beginning with the early observations of vesicle-like particles in plasma (1946) and the identification of platelet “dust” (1967). Key advances include: the demonstration of exosome release from multivesicular bodies and the coining of the term “exosome” in the 1980s; the recognition of their role in intercellular communication in the 1990s; and the transformative discovery of their nucleic acid cargo in the 2000s. The field’s maturation is marked by the establishment of ISEV (2011) and the MISEV guidelines (2014, 2018, and 2023). Recent years have witnessed accelerated clinical translation, evidenced by FDA-approved diagnostics, engineered therapeutics, and scalable manufacturing platforms. EVs: Extracellular vesicles; GMP: good manufacturing practice; mRNA: messenger RNA; FDA: Food and Drug Administration; RMAT: regenerative medicine advanced therapy; ARDS: acute respiratory distress syndrome; IVD: *in vitro* diagnostic; GPC1: glypican-1; MISEV2014: Minimal Information for Studies of Extracellular Vesicles 2014; MISEV2018: Minimal Information for Studies of Extracellular Vesicles 2018; MISEV2023: Minimal Information for Studies of Extracellular Vesicles 2023; ExoDx: exosome diagnostics test; EPI^TM^: ExoDx Prostate IntelliScore (a liquid biopsy test for prostate cancer); DC: dendritic cell; MVB: multivesicular body.

As interest in EV research continued to grow in the 2020s, efforts to standardize definitions, isolation methods, and characterization techniques have been underway to ensure consistency and reliability across studies. ISEV updated the MISEV guidelines to reflect evolving best practices in 2023^[24]^. The COVID-19 pandemic spurred investigations into exosomes as biomarkers and therapeutics for the disease^[47]^. In addition, the potential of EVs as drug delivery systems and therapeutic agents has also been extensively evaluated in the past decade. For example, in 2025, Kalluri *et al*. reported a Phase I clinical trial, NCT03608631, to evaluate engineered exosomes with Kras^G12D^-specific siRNA (iExoKras^G12D^) in pancreatic cancer, and demonstrated good safety and tolerability^[48]^. To date, approximately 500 clinical trials have been registered to evaluate the clinical potential of EV-based strategies, highlighting their promising future in biomedicine^[31,49]^. Figure 1 summarizes some milestone events in EV research.

**Figure 2:**
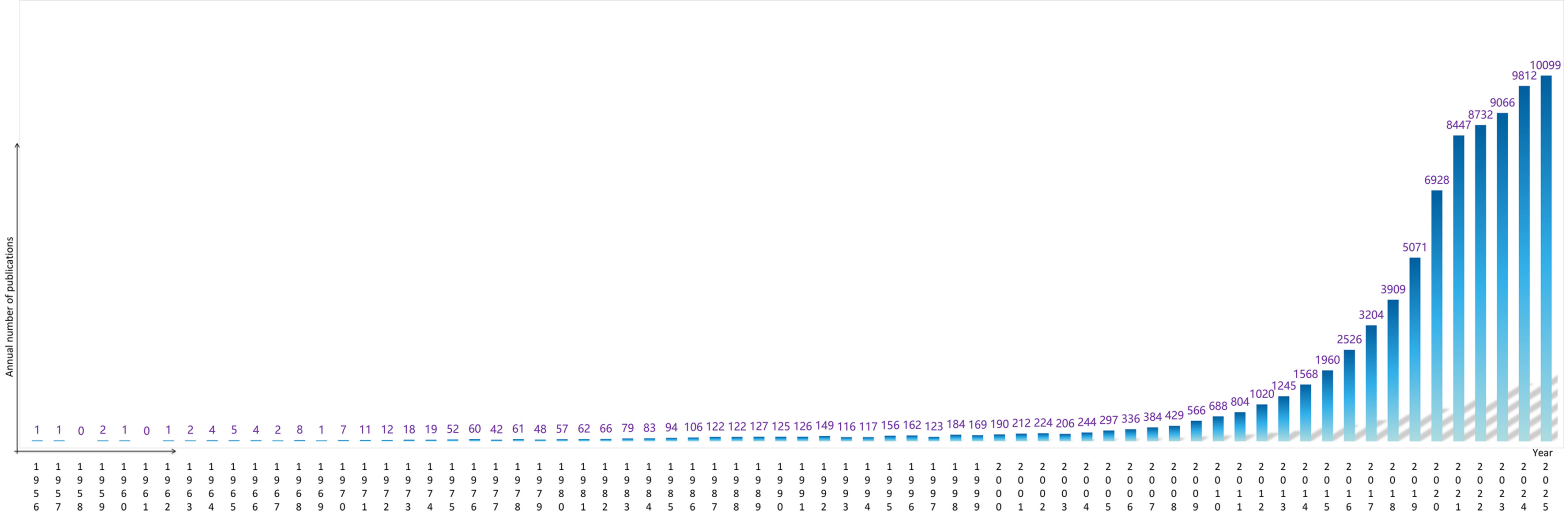
Annual number of publications on exosomes and EVs from 1956 to 2025. The histogram illustrates the growth trend of publications related to exosomes and EVs, retrieved from the PubMed database using the search query “exosome OR extracellular vesicle” (as of 2025). EVs: Extracellular vesicles.

In the early stages (1987-2000), publications on exosomes and EVs were limited. However, as research began to uncover their crucial roles in cell-cell communication and disease mechanisms - particularly their diagnostic and therapeutic applications - the field attracted growing interest. According to PubMed data, the number of related publications increased from 190 in 2000 to 688 in 2010. This growth accelerated dramatically thereafter, reaching 9,812 publications in 2024. As of 2025, approximately 10,099 studies have already been documented, reflecting a trend of rapid, near-exponential expansion [Figure 2]. This trend indicates that EV research has become a major focus in the biomedical field, attracting attention not only from researchers in basic science but also from the pharmaceutical and biotechnology industries, underscoring its broad translational and clinical potential.

### Guidelines on EVs

The rapid evolution of EV research has necessitated the development of standardized guidelines to ensure experimental rigor and reproducibility. ISEV has been at the forefront of this effort, releasing a series of MISEV guidelines. The inaugural MISEV2014 document provided the field with its first foundational framework^[39]^. This was significantly expanded in MISEV2018, which offered detailed recommendations on EV nomenclature, isolation and purification techniques, and characterization standards, strongly emphasizing comprehensive reporting to bolster research quality^[1]^. The most recent update, MISEV2023, not only incorporated advancements in methodological technologies but also provided guidance for translating EV research from basic science to clinical applications^[24]^. These consensus documents form a globally recognized, high-level framework that primarily focuses on experimental design, reporting criteria, and conceptual standardization.

The Chinese Society for Extracellular Vesicles (CSEV) issued its first group standards in 2021. Under the leadership of Professor Yang Wang, CSEV developed two national group standards “Small Extracellular Vesicles Derived from Human Pluripotent Stem Cells” (T/CRHA 002-2021)^[50]^ and “Small Extracellular Vesicles Derived from Human Mesenchymal Stem Cells” (T/CRHA 001-2021)^[51]^. These standards were officially released at the 6th Conference on Basic and Clinical Translational Research on Extracellular Vesicles in Shanghai in 2022, providing structured guidelines for the production and quality control of stem cell-derived EVs. The English versions of these documents were later published in the journal *iMed* in 2023^[52]^. In addition, in 2018, Zhang *et al*. successfully formed an expert consensus entitled “Consensus Statement on Exosomes in Translational Research and Clinical Practice”^[53]^. In 2023, the “2023-2024 Exosome Industry Development White Paper” was released, providing an in-depth analysis of the current state and future trends of the exosome industry. In the same year, the group standard titled “Application of Regenerative Medicine Exosome Technology in the Field of Aesthetic Dermatology” was officially launched by the China Association of Plastics and Aesthetics. In 2024, the Microbial Extracellular Vesicle Working Group of CSEV published the “Chinese Expert Consensus on the Nomenclature and Isolation of Bacterial Extracellular Vesicles”. These documents place greater emphasis on product-oriented technical specifications, manufacturing processes, and regulatory alignment, thereby operationalizing MISEV principles in real-world translational and industrial settings, in contrast to the primarily basic research-oriented focus of the MISEV series.

Concurrently, other professional groups and industry consortia have developed complementary standards to ensure the quality and controllability of EV-based products. Documents such as “Quality Control Standards for Mesenchymal Stem Cell Exosomes” and “Specification for Preparation and Testing of Human Mesenchymal Stem Cell Exosomes” establish reliable standard operating procedures for mesenchymal stem cell (MSC)-derived exosomes. The “General Technical Requirements for Extracellular Vesicles” provides a comprehensive guidance framework for the application of exosome technology, laying the groundwork for application standardization. Furthermore, “Guidelines for Clinical Translation and Commercialization of Extracellular Vesicles and Exosomes Based Therapeutics”^[54]^ and “Exosomes in Clinical Trial and Their Production in Compliance with Good Manufacturing Practice”^[55]^ emphasize the critical importance of standardization and regulatory compliance in clinical trials and commercial production.

Taken together, the MISEV guidelines provide an overarching international framework for EV research practice, while national and industry standards issued by regulatory agencies and professional societies in different regions translate these principles into concrete, context-specific requirements for manufacturing, quality control, and regulatory compliance. At the regulatory and standardization level, different countries are gradually converging in their overall approaches to EV-based therapeutics, although each country places emphasis on slightly different aspects. The U.S. Food and Drug Administration (FDA) primarily incorporates EVs into the regulatory framework for biological products and cell/gene therapies, focusing on risk control through the Investigational New Drug (IND) pathway, Good Manufacturing Practice (GMP)-compliant manufacturing, and systematic characterization centered on critical quality attributes (CQAs), such as particle size, cargo composition, and potency assays. The European Medicines Agency (EMA), in contrast, mainly builds on existing guidelines for Advanced Therapy Medicinal Products (ATMPs) and human cell-based medicinal products and, through scientific advice procedures and early-phase clinical trials, applies standardized donor and cell sourcing, rigorous control of upstream and downstream processes, and International Council for Harmonization (ICH)-compliant evaluation of quality, safety, and efficacy to EV products. Japan’s Pharmaceuticals and Medical Devices Agency (PMDA) relies on its regulatory framework for regenerative medicine and cellular therapies, with case-by-case review and long-term follow-up as notable features, emphasizing GMP, predefined CQAs, and a verifiable translational chain linking mechanistic preclinical data to the proposed clinical indications. By comparison, China’s framework, represented by CSEV documents and national expert consensus, is broadly aligned with the core principles of the FDA, EMA, and PMDA, likewise stressing cell source management, process controllability, and multidimensional EV characterization, while being distinguished by the relatively early issuance of EV-specific consensus documents that provide more detailed guidance on nomenclature, classification, and clinical translation pathways. Overall, these countries and regions, building on their respective regulatory traditions, are progressively converging toward a global trend of stringent quality control and evidence-based evaluation for EV products.

## BIOGENESIS, COMPOSITION, AND ABSORPTION OF EVs

### Mechanism of exosome formation and secretion

**Figure 3:**
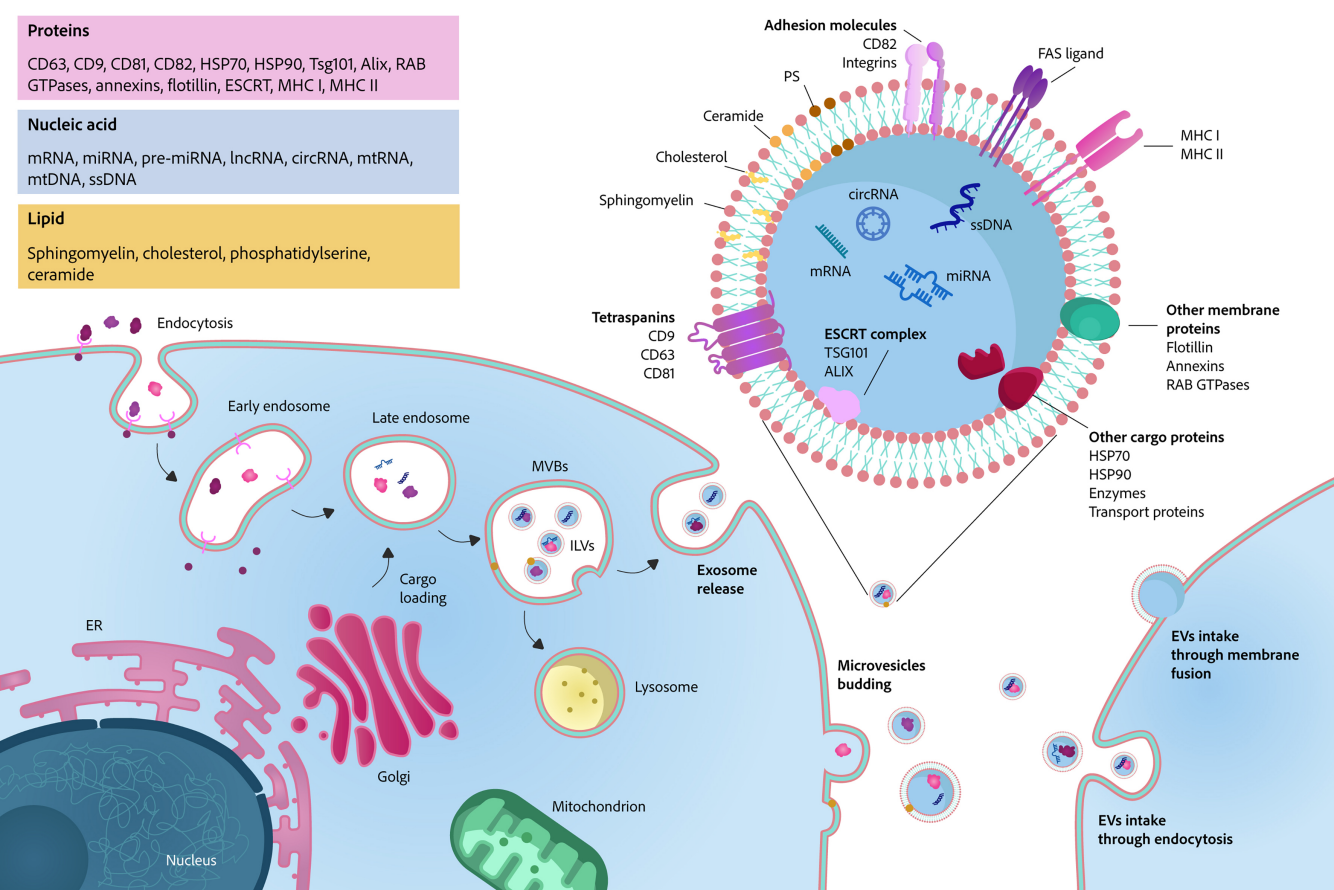
Schematic illustration of EV biogenesis, composition, and uptake. EVs are generated via two primary pathways: (1) the endosomal pathway, where early endosomes mature into late endosomes, which form ILVs within MVBs. These MVBs release ILVs as exosomes upon fusion with the plasma membrane; (2) direct budding from the plasma membrane, yielding microvesicles. EVs are composed of a lipid bilayer embedded with transmembrane proteins and enclosing a luminal cargo of diverse nucleic acids, proteins, and lipids. EVs can be internalized by recipient cells through endocytosis or direct membrane fusion. EVs: Extracellular vesicles; circRNA: circular RNA; ER: endoplasmic reticulum; ESCRT: endosomal sorting complex required for transport; FAS: Fas cell surface death receptor; HSP70: heat shock protein 70; HSP90: heat shock protein 90; ILVs: intraluminal vesicles; lncRNA: long non-coding RNA; MHC I: major histocompatibility complex class I; MHC II: major histocompatibility complex class II; mRNA: messenger RNA; miRNA: microRNA; mtDNA: mitochondrial DNA; PS: phosphatidylserine; ssDNA: single-stranded DNA; Tsg101: tumor susceptibility gene 101; MVBs: multivesicular bodies.

The biogenesis of EVs is not a singular, standardized process but rather an intricate, finely regulated, and context-dependent cellular activity [Figure 3]. At a conceptual level, EVs mainly arise from two interconnected routes: the endosomal pathway, in which intraluminal vesicles (ILVs) formed within MVBs are released as exosomes upon fusion with the plasma membrane, and direct outward budding from the plasma membrane, generating MVs. Among the heterogeneous populations of EVs, exosomes undergo a unique and well-characterized formation pathway within the endocytic system. Exosome biogenesis begins with the internalization of the plasma membrane and extracellular components into early endosomes (EEs), which progressively mature into late endosomes/MVBs. During this maturation, specific endosomal subdomains invaginate toward the lumen to generate ILVs. These ILVs either undergo degradation after fusion of MVBs with lysosomes, or are released as exosomes when MVBs fuse with the plasma membrane. The balance between these fates is tightly controlled by multiple trafficking and fusion machineries, including homotypic fusion and vacuole protein sorting (HOPS) and soluble *N*-ethylmaleimide-sensitive factor attachment protein receptors (SNARE) complexes^[56]^.

During endosome maturation, invagination of the endosomal membrane generates ILVs within the MVB lumen, which are subsequently released into the extracellular space as exosomes through fusion with the plasma membrane^[57]^. The biogenesis of ILVs is mainly regulated by the Endosomal Sorting Complex Required for Transport (ESCRT) complex^[58,59]^, tetraspanins^[60]^, and ceramide^[61]^. Beyond the classical ESCRT model, additional regulators such as the apoptosis-linked-gene 2 (ALG-2)-interacting protein X (ALIX)-syndecan-syntenin axis and specific lipid-modifying enzymes have been shown to coordinate ILV budding and cargo selection, providing a mechanistic link between membrane remodeling and signal-dependent exosome production.

Following ILV formation, specific regulators mediate the sorting of cargo, which ultimately defines the molecular composition of the released exosomes. For example, certain monoubiquitinated proteins can be recognized by ubiquitin-interacting-motif (UIM) of ESCRT-0, -I, and -II complexes and subsequently packaged into ILVs^[62-64]^. Prior to their final enclosure within MVB vesicles, the monoubiquitination modification can be removed from the cargo proteins by deubiquitinating enzymes such as Doa4^[65]^. In addition, some proteins are packaged into ILVs via ubiquitination-independent mechanisms. For example, interleukin-2 receptor (IL-2R)β can be sorted to the endosomes by docking onto the non-UIM domain of Vps27/Hrs complex^[66]^, whereas proteins with the KFERQ motif could be loaded into ILVs by lysosomal-associated membrane protein 2A (LAMP2A)^[67]^. Furthermore, autophagy-related pathways can regulate the loading of RNA-binding proteins (RBPs) into EVs^[68]^. Recent studies further suggest that phase separation of RBPs and membrane-associated condensates at the endosomal limiting membrane can cluster selected cargos before ILV budding, introducing a spatiotemporal layer of regulation to exosome biogenesis.

In contrast to proteins, the mechanisms mediating the loading of RNA and other cargos into ILVs are less well understood. MiRNA can be selectively loaded into ILVs by heterogeneous nuclear ribonucleoproteins A2/B1 (hnRNPA2B1) on the endosomal membrane^[69]^. Garcia-Martin *et al*. identified that miRNAs possess specific sorting codes that determine their preferential secretion in exosomes or cellular retention^[70]^. Additionally, RBPs are also considered key regulators in the selective sorting of various RNAs into EVs^[71,72]^. Moreover, epi-transcriptomic modifications such as N6-methyladenosine (m6A) and their “reader” proteins have been implicated in biasing particular transcripts toward exosomal export, thereby linking RNA modification states to EV-mediated communication.

After ILVs formation, MVBs fuse with the plasma membrane to release ILVs as exosomes, assisted by actin filaments, microtubules, motor proteins, and Rab family of small GTPases that activate these motors^[73]^. Some GTPases, including Rab27a, Rab5, and Rab4, play pivotal roles in regulating the formation, trafficking, and secretion of exosomes. In addition, environmental cues such as hypoxia, inflammatory cytokines, oncogenic signaling and metabolic stress can modulate Rab-dependent trafficking and MVB-plasma membrane fusion, dynamically tuning exosome release according to the cellular microenvironment.

### Biogenesis of MVs and apoptotic bodies

Beyond exosomes, there are other specific EVs such as MVs and apoptotic bodies. MVs, also known as ectosomes, are formed through the outward budding of the plasma membrane [Figure 3]. This process is orchestrated by intricate pathways, usually initiated by cellular activation or stress signals. In addition, multiple signals or processes, including calcium influx, cytoskeleton reorganization, and certain proteinases (e.g., floppases and scramblases), regulate MV/ectosome biogenesis^[74]^. At the sites of MV origin, specific molecular rearrangements occur, including alterations in lipid and protein composition and Ca^2+^ levels^[75,76]^, which collectively facilitate membrane budding. A hallmark feature of MVs is the externalization of phosphatidylserine on their surface - a characteristic typically absent in exosomes^[77]^. The elevated intracellular Ca^2+^ activates Ca^2+^-dependent enzymes such as calpain and gelsolin, which further remodel the membrane lipid architecture. Moreover, MVs are enriched in lipid rafts, and their formation can be inhibited by cholesterol deprivation^[78]^. The budding and release of MVs are also regulated by small GTPases, including members of the Rho family and adenosine diphosphate ribosylation factors (ARFs), which control cytoskeletal dynamics and vesicle secretion. Similar to exosomes, MVs carry diverse nucleic acids, including mRNA and DNA fragments. Recent studies have also linked MV shedding to plasma membrane repair pathways, indicating that cells can rapidly release MVs to remove damaged membrane domains and restore membrane integrity under mechanical or chemical stress^[79]^.

Apoptotic bodies constitute another major class of EVs, generated during the programmed cell death known as apoptosis. This process involves characteristic morphological changes such as plasma membrane blebbing, cell shrinkage, and chromatin condensation. During the final stages of apoptosis, large numbers of apoptotic bodies containing various cellular components are generated^[80]^. Routinely, these vesicles are rapidly engulfed by phagocytes to prevent the release of harmful intracellular contents and to avoid triggering inflammation or autoimmune responses in surrounding healthy tissues^[81]^. However, emerging evidence indicates that circulating apoptotic bodies can also be absorbed by other cell types, such as MSCs and CD8^+^ T cells, potentially mediating intercellular communication^[82,83]^. Beyond passive clearance, apoptotic cell-derived EVs have been shown to deliver autoantigens and immune-regulatory molecules, thereby influencing immune tolerance, resolution of inflammation, and, in some contexts, the development of chronic inflammatory and autoimmune diseases [Table 1].

**Table 1:** Major mechanisms and representative regulators involved in exosome biogenesis

**Major step/mechanism**	**Representative regulators**	**Principal roles in exosome biogenesis**	**References**
ESCRT-dependent ILV formation	ESCRT-0 (HRS, STAM), ESCRT-I (TSG101), ESCRT-II, ESCRT-III (CHMPs), and VPS4	Sequential recognition and clustering of ubiquitinated cargo, membrane budding into the endosomal lumen, and scission of ILVs	[58,59,73]
ESCRT-independent, lipid-driven ILV formation	Ceramide (nSMase2), sphingomyelinases, and cholesterol	Promote negative membrane curvature and microdomain formation, enabling ILV budding in the absence of canonical ESCRT components	[61,84]
Tetraspanin-enriched microdomains	CD9, CD63, CD81, and CD82	Organize tetraspanin-enriched microdomains, sort specific proteins and lipids into ILVs, and contribute to cargo selectivity	[85-87]
MVB transport along cytoskeleton	Actin filaments, microtubules, kinesin, and dynein motors	Mediate directional trafficking of multivesicular bodies toward the plasma membrane or lysosomes	[88]
Rab GTPase-mediated MVB trafficking and docking	Rab27a/b, Rab11, Rab31, Rab35, Rab5, and Rab4	Regulate MVB positioning, docking to the plasma membrane, and balance between exosome secretion and degradative routing	[89,90]
SNARE-dependent MVB-plasma membrane fusion	VAMP7, syntaxins, SNAP family proteins	Drive membrane fusion between MVBs and the plasma membrane, enabling the release of ILVs as exosomes	[91,92]
Regulation by oncogenic and signaling pathways	EGFR, RAS-MAPK, PI3K-AKT-mTOR, and p53	Oncogenic signaling pathways reshape endosomal trafficking, MVB fate, and exosome quantity/composition	[93,94]
Hypoxia-induced regulation	HIF-1α, HIF-2α Rab-dependent pathways	Hypoxia upregulates exosome production and alters cargo loading by modulating Rab GTPases, ESCRT components, and metabolic enzymes	[95]
Inflammatory and immune signaling	TNF-α, IL-1β, NF-κB, and pattern-recognition receptors	Inflammatory cues enhance exosome release and remodel their immunomodulatory cargo, contributing to intercellular inflammatory signaling	[96-98]
Metabolic and ER/oxidative stress	AMPK, mTOR, UPR sensors (PERK and IRE1), and ROS-related pathways	Cellular stress and metabolic rewiring alter MVB biogenesis, cargo sorting, and secretion as an adaptive mechanism to restore homeostasis	[99,100]

ESCRT: Endosomal sorting complex required for transport; ILV: intraluminal vesicle; HRS: hepatocyte growth factor-regulated tyrosine kinase substrate; STAM: signal-transducing adaptor molecule; TSG101: tumor susceptibility gene 101; CHMPs: charged multivesicular body proteins; VPS4: vacuolar protein sorting 4; nSMase2: neutral sphingomyelinase 2; MVBs: multivesicular bodies; Rab: Ras-associated binding protein; SNARE: soluble N-ethylmaleimide-sensitive factor attachment protein receptor; VAMP7: vesicle-associated membrane protein 7; SNAP: synaptosome-associated protein; EGFR: epidermal growth factor receptor; RAS: rat sarcoma; MAPK: mitogen-activated protein kinase; PI3K: phosphoinositide 3-kinase; AKT: protein kinase B; mTOR: mechanistic target of rapamycin; p53: tumor protein p53; HIF: hypoxia-inducible factor; TNF-α: tumor necrosis factor alpha; IL-1β: interleukin 1 beta; NF-κB: nuclear factor kappa B; AMPK: AMP-activated protein kinase; UPR: unfolded protein response; PERK: protein kinase R-like endoplasmic reticulum kinase; IRE1: inositol-requiring enzyme 1; ROS: reactive oxygen species.

In addition to classical membrane-bound EVs, a series of conceptual advances and recent discoveries has substantially reshaped our current understanding of the EV field. First, the “Trojan EV hypothesis” proposed in the early 2000s^[101]^ posits that retroviruses can hijack exosome biogenesis pathways to facilitate their dissemination, thereby providing an important theoretical framework to explain the evolutionary and structural similarities between EVs and enveloped viruses. In the context of the COVID-19 pandemic and rapid progress in viral vaccine research, this concept has regained prominence and further blurred the boundary between EVs and viruses. Second, a pioneering study^[102]^ in 2017 demonstrated that exosomal surface display of signal regulatory protein α (SIRPα) can block CD47 and enhance phagocytosis of cancer cells, representing a critical milestone in EV engineering. This work provided the first proof of concept that rational modification of exosomal surfaces can be directly translated into therapeutic efficacy, thereby inaugurating the strategy of “functional EV surface modification” and inspiring subsequent studies on “decoy EVs” and EV-based agonists/antagonists, particularly in the context of cancer immunotherapy. Third, the recent identification of non-EV nanoparticles, such as exomeres and supermeres, has revealed that these protein- and RNA-rich, non-membranous particles coexist with classical EVs and are functionally distinct from them^[103-105]^. The realization that some biological effects previously attributed to “exosomes” may in fact originate from these non-vesicular nanoparticles highlights a major limitation of current EV isolation and characterization approaches and underscores the need for more stringent fractionation and analytical standards in future studies. Collectively, these advances are driving a reappraisal of the fundamental biology of EVs and a more refined definition of their clinical translational potential.

Beyond these conceptual advances, multiple non-canonical secretion routes that depend on or bypass the ESCRT have now been identified, including ESCRT-independent, ceramide-driven budding, tetraspanin-enriched membrane microdomains, and unconventional trafficking mediated by Rab GTPases or SNAREs. These findings indicate that EV production is not merely a passive byproduct of endosomal maturation, but is instead dynamically and finely regulated by cellular state, metabolic cues, and organization of membrane microdomains. Likewise, cargo loading is increasingly recognized as an active and highly selective process rather than simple bulk encapsulation of cytosolic components. Specific RBPs, sequence or structural motifs within miRNAs/mRNAs, post-translational modifications of proteins, and lipid-protein interactions have all been shown to participate in directing cargo into defined EV subtypes. Overall, these discoveries support a model in which cells employ multiple, parallel biogenesis pathways for EVs and non-vesicular extracellular nanoparticles, each with distinct regulatory inputs and cargo-specifying mechanisms, thereby reshaping our understanding of how intercellular communication is encoded, sorted, and delivered under both physiological and pathological conditions.

### Components of EVs

EVs contain complex components, including but not limited to lipids, proteins, nucleic acids, and carbohydrates. The specific cargo profile of EVs is highly dynamic and is influenced by the cell of origin, its physiological or pathological state, and the extracellular microenvironment. To systematically catalogue this molecular diversity, several public databases have been established, such as Vesiclepedia (http://microvesicles.org/), exoRBase (http://www.exorbase.org/)^[45]^, and exRNA Atlas (https://exrna-atlas.org/).

#### Lipid components of EVs

The lipid bilayer of EVs exhibits a composition distinct from the plasma membrane of their parent cells, being notably enriched in cholesterol, sphingomyelin, glycosphingolipids (GSLs), and phosphatidylserine^[106]^. Cholesterol contributes to the increased rigidity and structural stability of the EV membrane^[107]^. Sphingomyelin and GSLs, including gangliosides, further stabilize membrane structure and participate in cell recognition and signal transduction processes^[108]^. These lipids contribute to the formation of microdomains within the EV membrane, which is essential for the docking and fusion processes during EV release and cellular uptake^[109]^. Beyond structural lipids, EVs carry bioactive lipids capable of modulating various signaling pathways^[110]^. For example, ceramides are directly involved in ESCRT-independent exosome biogenesis^[61]^, whereas exosomal leukotrienes can influence inflammatory responses of recipient cells. In addition, cancer-derived exosomes often exhibit altered lipid compositions and can transfer bioactive lipids into recipient cells, modulating tumorigenesis and progression^[110,111]^.

GSLs, a major subclass of glycolipids, are ubiquitous membrane components found in nearly all living organisms and are commonly found on EVs^[112]^. Gangliosides - a family of sialic acid-containing GSLs including monosialotetrahexosylganglioside (GM1), its precursor GM2, GM3, and disialodihexosylganglioside (GD3) - are prominently present in exosomes. Several gangliosides such as GM1 on EVs have been shown to regulate pathological processes involved in neurodegenerative disorders and cancers^[113,114]^.

#### Protein components of EVs

The EV membrane contains various membrane proteins. Integrins, for example, are present on EV surfaces and play a key role in directing EVs to specific recipient cells^[115]^. Major histocompatibility complex (MHC) molecules on the membrane of DC-derived EVs can activate T cells, underscoring the involvement of EVs in immune responses^[116]^. Additionally, tetraspanins, such as CD9, CD63, and CD81, are among the most abundant membrane proteins and are commonly used as markers for EV isolation and characterization. These proteins help organize membrane microdomains, facilitating the assembly of protein complexes essential for EV biogenesis and release. Other highly abundant membrane proteins in EVs, such as ESCRT proteins [e.g., Alix and tumor susceptibility gene 101 (TSG101)], have been reported to facilitate the inward budding of endosomal membranes during ILV formation^[3,117]^.

EVs carry a diverse array of bioactive proteins that influence recipient cell behavior. Cytosolic proteins such as HSP70 and HSP90, which mediate protein folding and stress response, are frequently observed in EVs^[118,119]^. Some metabolic enzymes in EVs, such as glyceraldehyde-3-phosphate dehydrogenase (GAPDH) and pyruvate kinase M2 (PKM2), can regulate metabolic reprogramming and cancer progression. Moreover, EVs can also transfer drug resistance-related proteins, including DNAJB8 [DnaJ Heat Shock Protein Family (Hsp40) Member B8]^[120]^, TrpC5 (human transient receptor potential canonical 5)^[121]^, RAB22A (RAB22A, Member RAS Oncogene Family)^[122]^, and programmed death-ligand 1 (PD-L1)^[123]^, thereby contributing to therapeutic resistance in recipient cells. EVs from MSCs (MSC-EVs) are notably enriched with proteins that promote tissue repair and regeneration. A comprehensive proteomic analysis by Anderson *et al*. identified 1,927 proteins in MSC-derived exosomes, including growth factors such as platelet-derived growth factor (PDGF), epidermal growth factor (EGF), and fibroblast growth factor (FGF), and components of the nuclear factor kappa B (NF-κB) signaling pathway^[124]^.

#### Nucleic acids in EVs

EVs contain both single-stranded and double-stranded DNA (ssDNA and dsDNA) with various sizes^[125]^. However, the mechanisms underlying the sorting of DNA into exosomes are not yet fully understood. This process may involve direct budding of nuclear or mitochondrial fragments into MVBs or the packaging of DNA through interactions with DNA-binding proteins and lipids^[126]^. EVs carrying mutant DNA sequences of KRAS (Kirsten rat sarcoma virus) and tumor protein p53 gene (*TP53*) can drive the malignant transformation of recipient cells^[127]^. Furthermore, viral DNA, such as herpes simplex virus and hepatitis B virus (HBV), could be encapsulated into the EVs of infected cells and be delivered to uninfected cells, aiding in the propagation of the infection^[128]^. A recent study proposes a new autophagy- and MVB-dependent, but exosome-independent, model for active secretion of extracellular DNA^[129]^. Compelling evidence from other studies, however, indicates that DNA is frequently detected in EV preparations and can be functionally significant^[130,131]^. This apparent contradiction may be reconciled by considering the heterogeneity of EV populations and diverse biogenesis pathways. For instance, autophagy-dependent secretory mechanisms (a process termed “EV secretion via autophagy”) have been shown to actively package cytoplasmic components, including fragmented nuclear or mitochondrial DNA, into double-membrane vesicles that are released extracellularly^[68]^. Similarly, distinct vesicle subtypes such as large oncosomes (shed from cancer cells) or arrestin domain containing 1 (ARRDC1)-mediated microvesicles (ARMMs) are also strongly implicated in the active sequestration and release of genomic and double-stranded DNA^[132]^. Consequently, the debate often centers not on the mere presence of DNA in EV preparations, but on its precise localization (intraluminal versus surface-adsorbed) and its biogenetic origin.

A wide variety of RNAs have been identified in EVs. In 2006, Ratajczak *et al*. revealed for the first time that MVs carried mRNAs that could be absorbed by recipient cells and translated into proteins^[33]^. In 2007, Valadi *et al*. reported that exosomes contain mRNA and miRNAs and can transfer them to recipient cells, thereby regulating their phenotypes^[14]^. Since then, an increasing number of RNA species have been observed in EVs. EVs are particularly rich in miRNAs, a type of small non-coding RNA (ncRNA) that regulates gene expression post-transcriptionally. For example, exosomal miR-21 derived from cancer cells promotes tumor progression by targeting multiple target genes in different recipient cells^[133,134]^. In addition to miRNAs, EVs also contain other ncRNAs such as lncRNAs, circRNAs and small nucleolar RNAs (snoRNAs)^[42,44,135]^. Glioblastoma-derived EVs transport tumor-specific mRNAs such as *EGF receptor variant III* (*EGFRvIII*) to promote angiogenesis and serve as diagnostic biomarkers^[136]^. Conigliaro *et al*. revealed that CD90^+^ liver cancer cells regulate endothelial cell (EC) phenotype via exosomal H19^[137]^. Moreover, EV RNAs show promise as biomarkers. Li *et al*. revealed that circRNAs are enriched in EVs and appear to be promising cancer diagnostic biomarkers^[42]^. Exosomal miRNAs, snoRNAs, and mRNA are also emerging as potential functional entities and promising candidate biomarkers^[135]^.

#### Mechanisms of EV absorption

EVs can be internalized by recipient cells via membrane fusion, a process mediated by several protein families, including SNARE proteins, Rab GTPases, and Sec1/Munc-18-related proteins (SM proteins)^[138]^. The primary mechanism for the uptake of EVs by cells is endocytosis, a multifaceted process encompassing macropinocytosis, phagocytosis, clathrin-mediated, caveolin-dependent, lipid raft-dependent, and clathrin/caveolin-independent endocytosis^[139]^. During this process, the contents of EVs must be released from the vesicle compartment into the cytoplasm; otherwise, they are transported to lysosomes for degradation. For instance, the cellular uptake of EVs derived from PC12 cells by other cells is regulated by clathrin-mediated endocytosis and macropinocytosis^[140]^. The extracellular signal-regulated kinase 1/2 (ERK1/2)-heat shock protein 27 (Hsp27) signaling pathway also mediates EV uptake, which is negatively regulated by Caveolin-1^[141]^.

Targeting mechanism of EVs depends on the interaction between EV surface proteins, lipids, or other components and receptors or ligands on target cells. This process is influenced by various external factors, enabling the specific targeting of EVs to recipient cells^[142,143]^. Protein-mediated targeting mechanisms are complex and diverse. Integrins, tetraspanins, and immune checkpoint molecules are among the most studied examples^[142]^. Tetraspanins (such as CD9, CD81, CD151) are highly expressed on the surface of EVs, and promote EV targeting and uptake through interactions with specific proteins on the surface of recipient cells. Immune checkpoint molecule-mediated targeting is a recently emerging area of research. PD-L1 on the surface of EVs binds to programmed death-1 (PD-1) on T cells, inhibiting T cell activity and immune response^[144]^. Lipid-mediated targeting is primarily executed by lipid rafts. Lipid rafts form stable microdomains on the exosome membrane, enhancing exosome targeting by interacting with lipid rafts or receptors on the target cell membrane. Additionally, the lipid molecule phosphatidylserine can bind to specific proteins (such as Tim4 and Tim1) on the surface of target cells, mediating exosome targeting^[145]^. Furthermore, collagen, fibronectin, and laminin in the extracellular matrix can interact with integrins and other binding proteins on the surface of EVs, regulating their distribution and targeting specificity^[146,147]^.

The uptake efficiency of EVs is influenced by various factors, including surface proteins and carbohydrates, pH levels, temperature, as well as EV size and concentration. For example, proteins, including integrins, tetraspanins, and MHC molecules, as well as carbohydrates present on the surface of EVs or recipient cells, can affect the binding efficiency of EVs to recipient cells^[148]^. Under low pH conditions, EVs exhibit increased rigidity and a higher sphingomyelin-to-ganglioside GM3 content, which facilitates the internalization of EVs into cells^[149]^. In contrast, low temperatures can reduce the activity of EV surface proteins, leading to a decrease in EV uptake^[150]^. Hypoxic conditions can regulate the generation of EVs and the expression of surface molecules, thereby affecting their targeting and uptake^[151]^.

## SEPARATION, PURIFICATION, AND CHARACTERIZATION OF EVs

### Separation and purification

**Figure 4:**
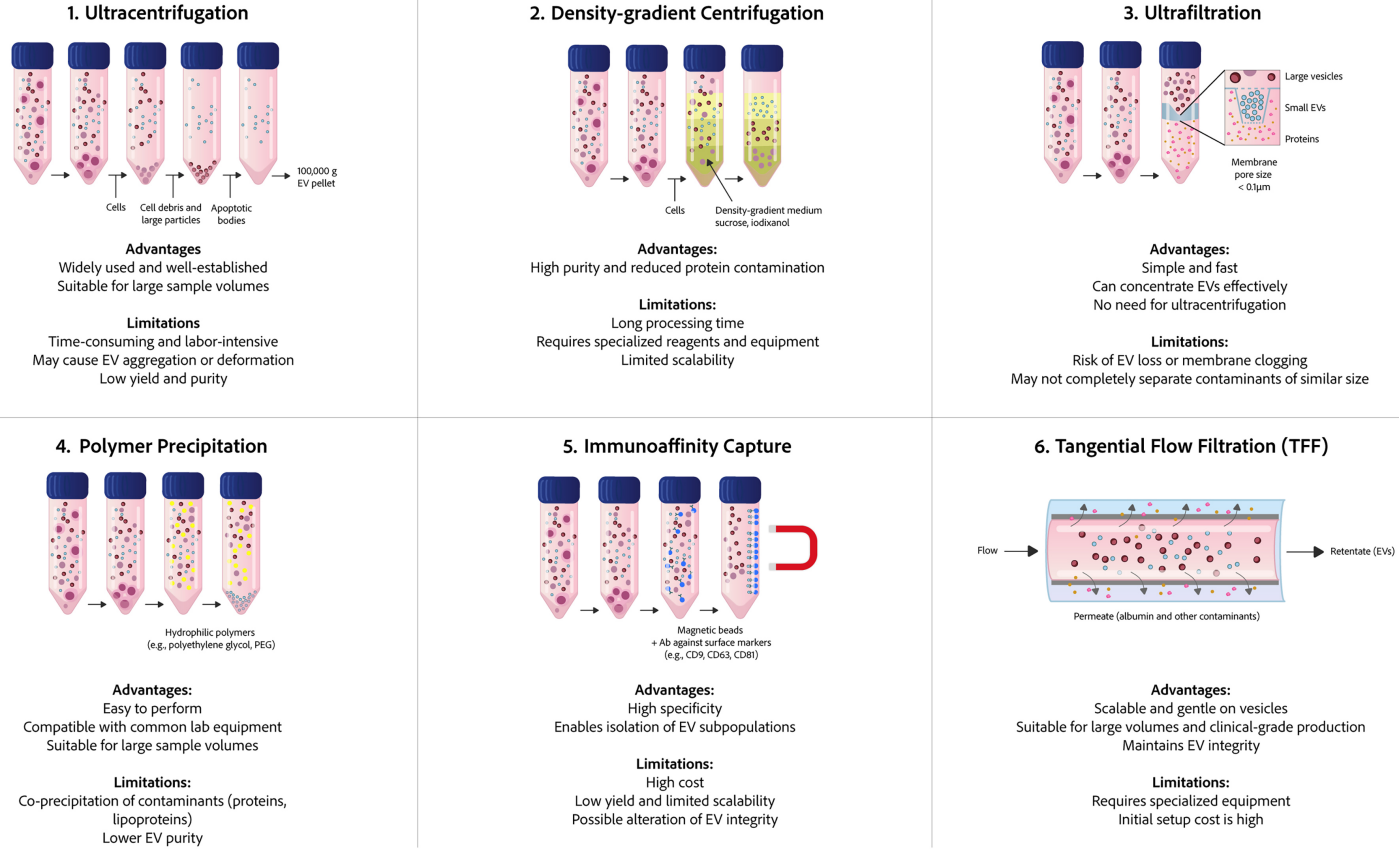
Comparison of six common methods for EV isolation. The figure provides a comparison of the fundamental processes, key benefits, and primary limitations associated with each method: ultracentrifugation, density-gradient centrifugation, ultrafiltration, polymer-based precipitation, immunoaffinity capture, and TFF. EVs: Extracellular vesicles; TFF: tangential flow filtration.

EVs represent a heterogeneous population of lipid-bilayer-enclosed vesicles that vary in size and contents. The physicochemical properties of EVs isolated by different separation methods can differ significantly, leading to considerable variability and poor reproducibility in EV research - major obstacles impeding EV basic research and clinical application. Based on the distinct physical and biochemical properties of EVs, a range of separation techniques have been developed, including ultracentrifugation, density gradient centrifugation, ultrafiltration, SEC, immunoadsorption, precipitation, and microfluidic-based separation technologies^[152]^. Figure 4 and Table 2 summarize the working principles, advantages, and disadvantages of commonly used separation methods for EVs^[153,154]^.

**Table 2:** Comparison of common EV isolation methods

**Method**	**Principle**	**Advantages**	**Disadvantages**	Purity^*^	Yield^*^	**Impact on EV integrity/activity**	**Processing time**	**Scalability**	**Clinical translatability potential**	**References**
UC	Sediments EVs based on size and density differences using high centrifugal force	Widely available, relatively low cost, yields relatively pure EVs, no special reagents required	Time-consuming, high shear force may compromise EV integrity, multiple steps, potential co-precipitation of impurities (proteins, nucleic acids)	++	++	Potential damage from high g-force	Long	Moderate (limited by rotor capacity)	High (current gold standard, but requires standardization)	[152,153,155]
Polymer-based precipitation	Uses polymers (e.g., PEG) to alter solubility, causing EVs to co-precipitate	Simple, fast, no special equipment, flexible sample volume, high yield, easy to scale up	Low purity, frequent co-precipitation of contaminants (proteins, nucleic acids, viruses), polymers may interfere with downstream analysis	+	+++	Polymers may affect activity/function	Short	High	Moderate (useful for pre-analytical steps in diagnostics, but purity is a bottleneck)	[156]
Ultrafiltration (UF)	Separates EVs by size using membrane filters with specific molecular weight cut-offs	Relatively fast, simple, can preserve biological activity	Membrane clogging reduces yield and efficiency, shear stress may damage EVs, not ideal for large volumes	+/++	++	Shear stress may cause rupture	Moderate	Low to moderate	Moderate (suited for small-scale research)	[157]
TFF	Sample circulates tangentially across a filter to minimize fouling, separating by size	Can handle large volumes, good reproducibility, minimal sample damage compared to UC/UF, suitable for scale-up	Longer processing time, more complex setup, prolonged processing may impair EV integrity	++/+++	+++	Relatively minimal, but risk with long processing	Long	High	High (particularly promising for industrial-scale production)	[158]
SEC	Separates by size as EVs migrate through porous gel beads at different rates	Gentle operation, high purity (minimal protein contamination), preserves EV integrity and biological activity well	Limited sample load, sample dilution, separation speed restricted by flow pressure/column size	+++	+/++	Very low (one of the gentlest methods)	Moderate	Low (difficult to scale with traditional columns)	Moderate (ideal for translational research requiring high purity, but low throughput)	[160]
Immunoaffinity capture	Uses antibodies (e.g., against CD9, CD63) to specifically capture EVs expressing specific surface markers	Very high purity and specificity, ideal for studying specific EV subpopulations	High cost, low throughput, may lose EV subsets lacking the target marker, requires elution step, antibodies may bind irreversibly	++++	+	Elution conditions (e.g., low pH) may affect	Moderate	Low	Low to Moderate (excellent for high-value target detection, but cost and complexity are barriers)	[159]
Microfluidics-based techniques	Integrates acoustic, electrophoretic, or electromagnetic forces on a chip to separate EVs based on physical/biological properties	Automated, low sample requirement, high throughput, high purity and efficiency, potential for integration with analysis	Early stage of development, complex device fabrication and operation, lack of standardization, currently small processing volumes	+++	Design-dependent	Can be minimal if well-designed	Short	Currently low, but high potential for parallelization	High (represents a future direction for point-of-care diagnostics and precision medicine, but needs further development)	[161,162]

^*^Low: +; moderate: ++; high: +++; very high: ++++. EV: Extracellular vesicle; UC: ultracentrifugation; PEG: polyethylene glycol; UF: ultrafiltration; TFF: tangential flow filtration; SEC: size exclusion chromatography; CD9: cluster of differentiation 9; CD63: cluster of differentiation 63.

Among these methods, ultracentrifugation is currently considered the standard method for EV purification because of its convenience, low cost, and ability to yield relatively pure EVs. However, the entire process of ultracentrifugation is quite time-consuming, and the prolonged high-force centrifugation may compromise EV integrity, potentially altering their morphology and biological activity. In addition, there are still some impurities in the purified EVs even after multiple centrifugation steps^[155]^. In contrast, precipitation is a simple and fast method that does not require specialized equipment, enabling high yield and easy scalability^[156]^. A major drawback, however, is the frequent co-precipitation of contaminants such as proteins, nucleic acids, and other extracellular particles (e.g., viruses), which substantially reduces the purity of the isolated EVs.

Ultrafiltration isolates EVs based on the particle size using membrane filters. Although this one-step approach is faster than ultracentrifugation, the shear stress induced by applied pressure may damage EVs, and filter clogging due to accumulated particles may reduce yield and increase processing time^[157]^. As an alternative, tangential flow filtration (TFF) has been developed to minimize membrane fouling by circulating the sample tangentially across the filter^[158]^. TFF represents a promising method to handle large volumes of samples with good reproducibility and minimal damage to samples compared with ultracentrifugation and other conventional filtration methods. However, the longer processing time of TFF may adversely affect EV integrity and functional properties.

Immunoaffinity capture isolates EVs using antibodies against specific surface markers (e.g., CD9, CD63, and ALIX), offering high purity and selectivity. However, its widespread application is limited by high cost, low throughput, the need for additional elution steps, and the absence of a universal EV marker - which may result in the loss of certain EV subpopulations during isolation^[159]^. The precipitation method, in contrast, relies on polymers that interact with water molecules to reduce EV solubility, facilitating the sedimentation of vesicles. While commonly used, this approach lacks the specificity of immunoaffinity techniques. SEC, another commonly employed technique, serves as a gentler alternative to ultrafiltration and ultracentrifugation. It demonstrates significant advantages in yielding high-purity EVs with minimal protein contamination, making it particularly suitable for downstream analyses^[160]^. However, the duration of SEC is limited by flow pressure. More recently, microfluidics-based technologies have attracted increasing attention by integrating emerging techniques such as acoustic, electrophoretic, and electromagnetic manipulations. These systems offer automation, low sample requirements, and high-throughput processing while maintaining high purity and efficiency^[161,162]^. Despite these promising features, microfluidics remains at an early developmental stage and requires further optimization. Key challenges, such as the complexity of device fabrication and operational procedures, represent urgent priorities before this technology can be translated into clinical practice.

In addition to the aforementioned methods, researchers have also explored new technologies, including plasmon resonance biosensors^[163]^, rotary ultrafiltration, nanolipid probe systems, microfluidic platforms, thermoacoustic fluid separation techniques based on cholesterol content, and immunomodified superparamagnetic nanoparticles^[164]^, to provide new avenues for quick, efficient, and high-purity EV separation, thereby facilitating their downstream applications. For example, Chen *et al*. developed a highly efficient exosome isolation platform, EXODUS, that allows automated label-free purification of exosomes from varied biofluids^[165]^.

The comparison of common EV isolation methods is summarized in Table 2. However, none of these existing methods simultaneously offers the desired speed, simplicity, and efficiency while ensuring that the morphology, purity, yield, and biological activity of the extracted EVs meet the standards required for subsequent experiments. Integrating current extraction methods can help reduce sample impurities. Further investigation into the physicochemical properties of EVs will also contribute to improved isolation purity.

EVs exhibit good stability and are convenient to store. Standard preservation methods for EVs involve resuspending them in phosphate-buffered saline (PBS) and storing at -80 °C for up to one year or at -20 °C for at least six months, with no obvious changes in morphology or biological properties. Moreover, an acidic environment with low pH has been shown to promote stable storage of EVs and enhance their yield^[166]^.

### Characterization of EVs

EVs are typically characterized by their morphology, particle size distribution, and biochemical composition. Current methods for EV analyses primarily include microscopic image observation for morphology, particle size analysis, and surface marker identification.

Electron microscopy and Atomic Force Microscopy (AFM) are basic tools for the morphology analyses of EVs. Scanning electron microscopy (SEM), commonly used to examine EV morphology and size, employs electromagnetic lenses to focus an electron beam on the sample surface, thereby obtaining its surface information^[167]^. SEM has many advantages, including a large depth of field, a wide magnification range, and relatively simple sample preparation.

Transmission electron microscopy (TEM) has higher resolution (below 1 nm) than SEM. However, due to the high vacuum environment required for TEM, biological samples must undergo fixation and dehydration, which may affect the size and morphology of EVs^[168]^. The tedious sample preparation process also results in longer analysis time. Under the microscope, exosomes often exhibit a cup-shaped, bilayer membrane-enclosed structure, but this morphology may be an artifact produced during the drying process^[169]^.

AFM, a type of scanning probe microscopy, characterizes surface structure and properties by measuring interactions between a sample’s surface atoms and a microcantilever tip^[170]^. It can achieve sub-nanometer resolution. Given its high sensitivity, EVs must be immobilized on an ultra-smooth substrate such as mica. Antibodies can be employed to attach EVs to this surface, allowing for the acquisition of biochemical information. However, the efficiency of antibody-mediated EV attachment remains unclear, limiting accurate assessment of EV concentration and morphology.

Light scattering techniques are commonly employed to determine the size distribution of EVs^[171]^. Dynamic light scattering (DLS) correlates Brownian motion with particle size, enabling detection in the range of 1 nm to 6 μm^[172]^. However, DLS tends to overestimate EV size due to interference from hydration layers and other interfacial effects. Nanoparticle tracking analysis (NTA) is a commonly used tool to characterize both EV size and concentration by tracking the motion of individual particles and calculating their mean square displacement^[173]^. Tunable Resistance Pulse Sensing (TRPS) is a more recent single-particle, *in situ* characterization technique^[174]^ that has been successfully used to characterize EVs. Unlike DLS and NTA, TRPS does not rely on Brownian motion, requires smaller sample volumes, offers faster measurement, and can simultaneously determine particle size, concentration, and zeta potential of EVs.

Although these techniques are reliable, semi-quantitative, and require minimal sample volumes, they cannot identify the cellular origin of EVs or distinguish them from lipoprotein complexes, membrane fragments, or other cellular components. Therefore, they should be combined with more qualitative approaches, particularly electron microscopy, for accurate EV identification.

EVs carry many specific proteins that can be identified immunologically. Western blot is the most commonly used method for EV protein detection and characterization due to its strong specificity and broad usability. Enzyme-linked immunosorbent assay (ELISA) is another frequently utilized method, offering higher quantitative accuracy than Western blot, though it cannot provide protein molecular weight information. In addition, colorimetric-based methods have been applied for rapid detection of EV surface proteins, enabling characterization of subtle differences within minutes^[175]^.

Flow cytometry (FCM) is a high-throughput, multi-parameter technique recently adapted for EV analysis^[176]^. Since the size of most EVs is below the detection limit of conventional FCM, nano-FCM has been developed to detect vesicles as small as 100 nm. Fluorescent labeling of EVs enables their detection by FCM. However, the “swarm effect” can interfere with accurate particle discrimination from background noise, complicating precise quantification^[177]^. An alternative approach uses latex microspheres conjugated with EV protein-specific antibodies. Captured EVs can be further labeled with fluorescent antibodies for surface antigen analysis, though the exact number of EVs bound per microsphere remains uncertain. Recently, improved FCM methods have been developed for EV quantification and single-vesicle analysis^[178,179]^.

For more intuitive visualization of EV morphology and surface proteins, EVs can be labeled with lipophilic dyes [e.g., Paul Karl Horan (PKH) 67] and DiD (1,1’-dioctadecyl-3,3,3’,3’- tetramethylindodicarbocyanine, 4-chlorobenzenesulfonate salt) or fluorescent antibodies and observed using confocal laser scanning microscopy (CLSM).

### *In vivo* EV imaging

*In vivo* imaging of EVs has several typical application scenarios: (1) monitoring the biogenesis, uptake, and biodistribution of EVs; (2) analyzing the pharmacokinetic characteristics of EV-based therapeutic platforms; and (3) detecting diseases via disease-related EVs. However, *in vivo* EV imaging is particularly challenging due to their small size, the lack of distinctive markers distinguishing them from host cells, and their rapid clearance *in vivo*. Advances in EV labeling and molecular imaging techniques have facilitated the visualization of EVs in living organisms. EV membrane components such as lipids and proteins are ideal anchoring media for contrast agents, owing to the natural production process of EVs through endogenous budding and membranous release. Strategies for *in vivo* EV imaging mainly fall into two categories: (1) *in vivo* targeted labeling of EVs inside the body; and (2) *in vitro* isolation and labeling of specific EVs^[180]^. Although the *in vivo* labeling approach more closely reflects native conditions, its major limitation is the difficulty in distinguishing EVs from cells which has limited its widespread application. Therefore, most EV tracking strategies rely on *ex vivo* labeling, performed either directly on isolated EVs or indirectly on their parent cells. These pre-labeling strategies are effective and controllable. Typically, EVs with specific accumulation behavior at lesion sites are selected for labeling, and certain ligands may be engineered on EVs to enhance disease-specific accumulation^[181]^.

#### Direct EV labeling

Agents that can release different signals are loaded onto EVs directly via biological or physical approaches, making this strategy widely used for *in vivo* EV imaging. Some lipophilic molecules, including the commercial PKH series (PKH26 and PKH67), carbocyanine dyes [DiO (3,3’-dioctadecyloxacarbocyanine perchlorate), DiI (1,1’-dioctadecyl-3,3,3’,3’-tetramethylindocarbocyanine perchlorate), and DiR (1,1’-dioctadecyl-3,3,3’,3’-tetramethylindotricarbocyanine iodide)], and other lipid-conjugated fluorescent probes [e.g., cholesterol- or DSPE (1,2-distearoyl-sn-glycero-3-phosphoethanolamine)-linked dyes], can be attached to or fused with the EV membrane via direct incubation. For example, DSPE-PEG-Cy7 (DSPE-polyethylene glycol with conjugated cyanine 3) was loaded onto exosomes derived from urine samples of prostate cancer patients for tumor monitoring^[182]^. Electroporation is another commonly used technique for loading contrast agents, especially for nanoparticles, into EVs^[183]^. Although efficient, electroporation can cause unexpected membrane damage and cargo leakage, potentially compromising EV integrity. Alternative internalization strategies, such as membrane fusion or transporter-mediated endocytosis, have also been explored^[184]^. For example, glucose-coated gold nanoparticles (GNPs, ~5 nm) were internalized into EVs via glucose transporter type 1 (GLUT-1)^[185]^. The GNPs were further applied in the *in vivo* tracking of MSC-EVs, which displayed distinct migration patterns in different brain pathologies. This specific targeting and accumulation behavior endows MSC-EVs with great potential in diagnosis and evaluation of brain diseases^[186]^. Overall, direct labeling is simple, low-cost, and efficient, making it a suitable and reliable approach for various EVs, especially those collected from bodily fluids with unknown cellular origins.

#### Indirect EV labeling via parent cells

Indirect labeling has been widely demonstrated to be an effective strategy, offering advantages in long-term stability and minimal off-target effects. By modifying or genetically engineering parent cells, signal components or functional groups can be subsequently incorporated into the secreted EVs. A more commonly used indirect labeling method involves co-expressing exosomal marker proteins with fluorescent proteins [such as green fluorescent protein (GFP), enhanced GFP (eGFP), and red fluorescent protein (RFP)] or bioluminescence reporters. CD63, the C1C2 domains of lactadherin, and palmitoylation sequences were frequently selected as targets for EV genetic engineering^[187]^. For example, Hikita *et al*. employed a bioluminescence resonance energy transfer (BRET)-based reporter, Antares2, to monitor the homing behaviors of prostate cancer-derived exosomes, achieving long-term and deep-tissue *in vivo* imaging of EVs^[188]^. However, the process of engineering recombinant proteins is often tedious and time-consuming, which may affect EV biogenesis and cellular recognition processes.

Alternatively, some chemically active groups can be introduced onto EV membrane by metabolic labeling. Phospholipids are regarded as ideal substrates, as they minimize functional interference compared to recombinant fusion proteins. Zhang *et al*. developed a phospholipid-based biorthogonal labeling for exosomes. An unnatural choline analogue bearing an azide was incubated with parent cells, incorporated into cell membrane, and subsequently transferred to the EV membranes. The azide groups then reacted with aza-dibenzocyclooctyne (DBCO)-conjugated fluorescent dyes, enabling stable imaging of EVs^[189]^. A similar strategy has also been applied using azido-sugars, which are metabolically incorporated into exosomal glycans^[190]^. This *in situ* bioorthogonal labeling strategy offers high-yield, operational simplicity, and minimal impact on EV function. In a comparative study evaluating direct and indirect labeling in an acute kidney injury mouse model, indirect imaging demonstrated superior specificity and longer signal retention^[191]^.

#### In vivo EV imaging techniques

Reported *in vivo* tracking techniques for EVs include optical imaging, nuclear imaging, tomographic imaging, and photoacoustic imaging. Optical imaging mainly consists of fluorescent imaging and bioluminescent imaging, which primarily differ in whether an external excitation module is required. Bioluminescent signals generated from the luciferase enzyme-substrate reaction offer higher sensitivity and lower background noise compared to fluorescent imaging. However, the need for substrate injection and the limited half-life of the substrate restrict the broader application of bioluminescent imaging^[192]^. Optical imaging is relatively simple and low-cost, but it suffers from limited penetration depth and imaging resolution. Though imaging quality can be improved using long-wavelength light signals, achieving sufficient spatial resolution remains challenging compared to other imaging techniques. Nuclear imaging techniques, such as single-photon emission computed tomography (CT) (SPECT) and positron emission tomography (PET), are often combined with anatomical imaging via tomography to determine EV location. The deep tissue penetration of nuclear imaging enables it to be applicable to any organ. Nuclear agents are typically incorporated into EVs through direct labeling. For instance, ^99m^Tc- and ^125^I-based EV imaging has been widely reported in preclinical studies^[193]^.

Magnetic resonance imaging (MRI) and CT are leading clinical imaging techniques and can also be applied to EV tracking with the assistance of metal nanoparticles such as GNPs^[194]^. Photoacoustic imaging has emerged recently as a promising tool for EV tracking. Unlike conventional ultrasound imaging, photoacoustic imaging employs contrast agents with optical absorption properties, which can be loaded onto EVs via direct or indirect approaches. This approach combines the spectroscopic specificity of optical imaging with the high spatial resolution of ultrasound imaging^[194]^.

Taken together, the tissue penetration and homing properties of EVs make them effective tools for *in vivo* disease monitoring, particularly in inflammation, tumor, and neurological diseases. Notably, different labeling and imaging approaches have their own advantages and limitations, depending on the application scenarios. Optical imaging is highly suitable for localized observation in specific regions, while various tomographic imaging techniques are more suitable for whole-body monitoring.

## DIVERSE FUNCTIONS AND MECHANISMS OF EVs IN BIOLOGICAL SYSTEMS

**Figure 5:**
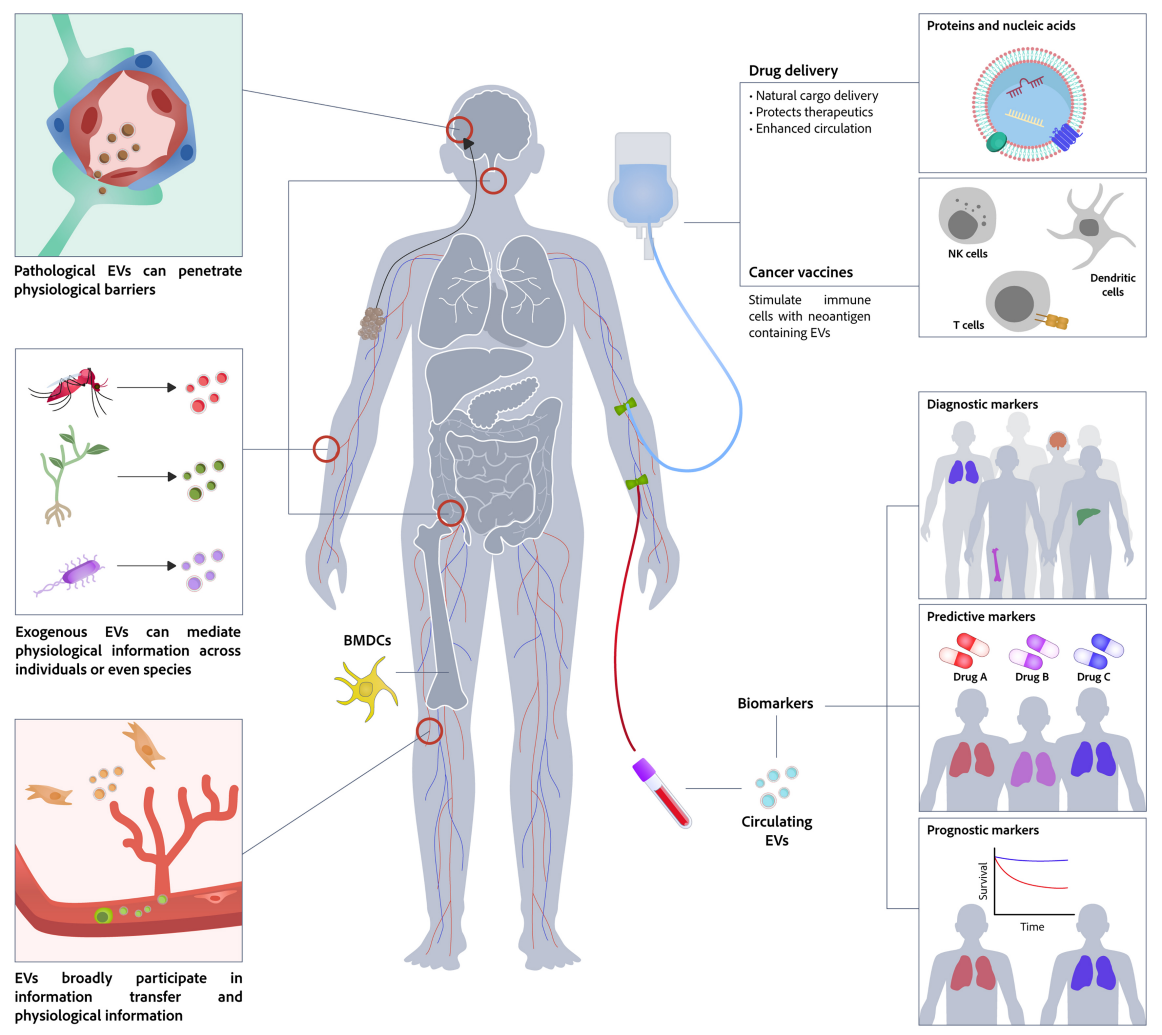
Physiological and translational functions of EVs. As crucial mediators of intercellular communication, EVs facilitate information exchange not only within an organism but also between individuals and across species. Their innate properties make them ideal candidates for diverse translational applications, including engineered drug delivery systems with improved targeting, novel vaccines that stimulate antitumor immunity, and sensitive biomarkers for liquid biopsy-based disease diagnosis and monitoring. EVs: Extracellular vesicles; NK cells: natural killer cells; BMDCs: bone marrow-derived dendritic cells.

EVs can modulate recipient-cell functions and phenotypes through their cargo, thereby influencing diverse cellular processes under both physiological and pathological conditions. Growing evidence indicates that EVs play important roles in various human diseases, particularly cancer, central nervous system (CNS) disorders, CVDs, and metabolic diseases. Moreover, EVs can serve as diagnostic biomarkers that reflect the status of their cells of origin and can also be used as therapeutics or drug-delivery vehicles for disease detection and targeted treatment [Figure 5].

**Figure 6:**
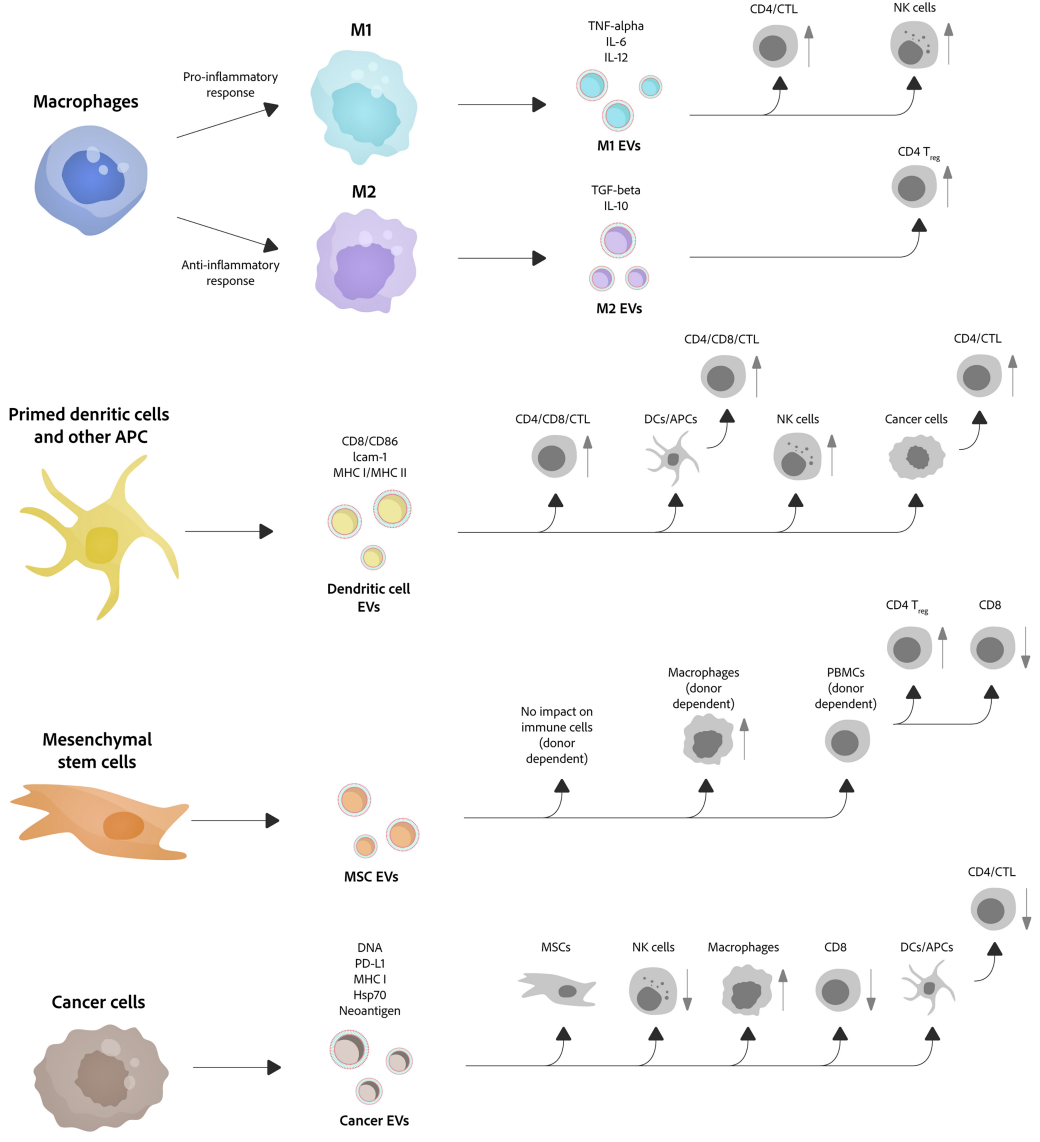
Immunomodulatory functions of EVs. EVs derived from immune cells, MSCs, and cancer cells exhibit diverse immunomodulatory effects. Macrophage-derived EVs display polarization-dependent functions: pro-inflammatory M1-EVs activate CD4^+^ T cells, CTLs, and NK cells, whereas anti-inflammatory M2-EVs foster regulatory T cell (Treg) generation and immunosuppression. Additionally, DC-derived EVs, which express CD8, CD86, ICAM-1, and MHC I/II complexes, can transfer antigen-presenting capacity to other APCs, thereby activating CD4^+^ and CD8^+^ T cells and NK cells to enhance antitumor immunity. EVs derived from mesenchymal stem cells modulate the functions of macrophages, PBMCs, and T-cell subsets in a donor- and context-dependent manner. Conversely, tumor-derived EVs carry molecules such as DNA, PD-L1, MHC I, Hsp70, and neoantigens, which can manipulate various immune cells to drive immune evasion and tumor progression. M1 EVs: M1 macrophage-derived extracellular vesicles; M2 EVs: M2 macrophage-derived extracellular vesicles; APC: antigen-presenting cell; EVs: extracellular vesicles; MSC-EVs: mesenchymal stem cell-derived extracellular vesicles; MSCs: mesenchymal stem cells; PBMCs: peripheral blood mononuclear cells; DCs: dendritic cells; CTL: cytotoxic T lymphocyte; T_reg_: regulatory T cell; NK cells: natural killer cells; DNA: deoxyribonucleic acid; PD-L1: programmed death-ligand 1; MHC I: major histocompatibility complex class I; Hsp70: heat shock protein 70.

EVs originate from a wide range of sources - including immune cells, stem cells, tumor cells, plants, and microbes - and mediate intercellular communication by carrying proteins, nucleic acids, lipids, and small molecules. They regulate immune responses, tissue repair, tumor progression, cross-kingdom signaling (e.g., plant-animal and microbe-host interactions), and pathogen transmission. Immune cell-derived EVs can activate or suppress immune responses and participate in antigen presentation; stem cell-derived EVs promote regeneration and immune modulation; tumor-derived EVs drive tumor growth, immune evasion, and drug resistance; and plant- and microbe-derived EVs play important roles in nutrition, gut homeostasis, antiviral defense, and pathogenesis [Figure 6].

### EVs from immune cells

Similar to immune cells, EVs derived from immune cells can exert either immune-activating or immunosuppressive functions. EVs originating from immune cells, including T cells, B cells, DCs, and natural killer (NK) cells, regulate immune functions through multiple mechanisms^[195]^. Early studies found that B cell-derived EVs carrying MHC class II can directly stimulate CD4^+^ T cell clones, indicating the role of EVs in antigen presentation^[196]^. EVs secreted by DCs contain MHC-I and MHC-II molecules and activate specific T cells by binding to T-cell receptors (TCRs)^[197]^. T cell-derived EVs contain TCRs and co-stimulatory molecules, which can interact with antigen-presenting cells (APCs) to enhance T cell immune responses. NK cells regulate the function of APCs through cytokine secretion and direct cytotoxic effects. In contrast, EVs secreted by regulatory T cells (Tregs) contain inhibitory molecules [e.g., transforming growth factor-β (TGF-β) and interleukin (IL)-10], which suppress the activity of effector T cells and other immune cells, maintaining immune tolerance and preventing autoimmunity. Macrophage-secreted EVs contain various cytokines, including IL-1β and tumor necrosis factor (TNF)-α, which can regulate local inflammatory responses and immune cell recruitment^[198]^. Interestingly, EVs derived from host immune cells can also help prevent viral infections by activating immune cells and inducing antiviral adaptive immune responses^[199]^.

### Stem cell-derived EVs

EVs derived from stem cells exhibit strong tissue repair and regeneration capabilities. For instance, MSC-EVs can promote the regeneration of cartilage, bone, and cardiomyocytes^[200]^. EVs from neural stem cells (NSCs) contain neurotrophic factors and miRNAs, promoting the growth and repair of neurons^[201]^. EVs derived from multiple stem cell types can promote neovascularization, improve blood supply, and enhance myocardial repair^[202]^. Owing to the diverse capabilities of stem cell-derived EVs, they possess significant potential in clinical applications. For instance, EVs can alleviate neuroinflammation and protect neurons by delivering specific miRNAs and proteins, offering potential therapeutic effects for neurodegenerative diseases (NDs)^[203]^. Furthermore, natural or engineered stem cell-derived EVs also show promise in treating inflammatory and immune-related diseases by modulating immune cell functions^[204]^. Additionally, stem cell-derived EVs can carry antioxidant components to regulate cellular aging processes in recipient cells^[205]^.

### Tumor cell-derived EVs

Cancer cell-derived EVs carry growth factors and cytokines [e.g., TGF-β, IL-6, and vascular endothelial growth factor (VEGF)] that promote cancer progression by activating multiple oncogenic signaling pathways, inducing tumor proliferation, epithelial-to-mesenchymal transition, drug resistance, and angiogenesis^[206]^. They also facilitate tumor immune evasion through various mechanisms. For instance, exosomal PD-L1 can bind to PD-1 on T cells, inhibiting T cell activity and promoting immune evasion^[207]^. Additionally, tumor EVs can promote the expansion and function of Tregs, suppressing the activity of effector T cells and enhancing immunosuppressive effects^[208]^. Furthermore, tumor EVs can remodel the tumor microenvironment (TME) by reprogramming stromal cells^[209]^ and altering the metabolic state of the TME via the transfer of metabolism-related enzymes and metabolites^[210]^. Interestingly, recent studies highlight the key role of tumor EVs in chemoresistance, posing both a therapeutic challenge and a potential target for intervention^[122,211]^.

### Plant-derived EVs

Plant-derived EVs (PDEs) are morphologically and biogenetically comparable to mammalian EVs^[212,213]^. Given the close relationship between plant-based foods and human health, dietary uptake of PDEs may represent a novel mechanism for cross-kingdom regulation. PDEs exhibit extensive regulatory functions in mammalian cells through diverse mechanisms, particularly through miRNA-mediated regulation^[214]^. They can be absorbed by intestinal cells in humans and animals via endocytosis and transcytosis. PDEs can either enhance or suppress immune responses by modulating macrophages and T cells via inflammatory mediators^[215]^.

Moreover, PDEs can inhibit tumor cell proliferation and induce apoptosis. For example, PDE miR-159 inhibited breast cancer progression by targeting transcription factor 7 (TCF7)^[216]^. Specific proteins in ginseng-derived EVs can activate apoptotic pathways in cancer cells, promoting cell death^[217]^. PDEs also promote gut health by regulating the gut microbiota and protecting the intestinal barrier. For instance, broccoli-derived EVs help maintain gut homeostasis by providing nutrients necessary for the growth of beneficial microbiota^[218]^. Proteins in citrus EVs can enhance the tight junctions of intestinal epithelial cells, thereby reinforcing the intestinal barrier^[219]^. In addition, PDEs are also involved in antiviral defense^[220]^. In recent years, the roles of PDEs in maintaining tissue homeostasis and organismal integrity have been increasingly recognized, highlighting PDEs as promising biotherapeutic tools for both plant and mammalian diseases^[221]^.

### Microbe-derived EVs

Outer membrane vesicles (OMVs) are EV-like vesicles produced by Gram-negative bacteria^[222]^. Other microorganisms, such as fungi and viruses, also release EVs^[223,224]^. These microbe-derived EVs play important roles in physiological functions and pathological processes in both the microorganisms themselves and their host. In recent years, with the advancement of nanotechnology and molecular biology, the structure and functions of microbial EVs have gradually been elucidated^[225]^. Similar to mammalian EVs, microbial EVs carry various bioactive molecules including proteins, nucleic acids, and lipids, participating in intercellular communication, microenvironment regulation, and host-pathogen interactions^[226]^.

Microbial EVs play a dual role in infection and immunity^[227]^. On the one hand, they act as carriers of pathogenic factors, delivering toxins and virulence-associated molecules that promote microbial invasion and colonization, thereby exacerbating infection. On the other hand, these vesicles can activate the host immune system, inducing both innate and adaptive immune responses. Notably, components such as lipopolysaccharides (LPS), outer membrane proteins, and small RNAs found in bacterial EVs are considered critical signals for modulating host defense mechanisms. Moreover, microbial EVs are involved in biofilm formation and bacterial quorum sensing, playing an indispensable role in maintaining microbial ecosystem balance^[228,229]^.

Recent studies have shown that virus-released EVs also mediate the crosstalk between viruses and host cells. Many viruses, such as human immunodeficiency virus (HIV), HBV, and hepatitis C virus (HCV), can utilize the exosome biogenesis mechanism of host cells to package viral particles into EVs, facilitating intercellular spread^[223]^. Moreover, viruses can directly transmit their RNA or proteins to host cells through EVs, promoting viral replication and spread^[230]^.

Microbial EVs hold great application potential, especially in disease diagnosis, vaccine development, and drug delivery. Due to their ability to carry specific molecules and their natural cell-targeting properties, researchers are exploring the use of microbial EVs as biomarkers to improve detection sensitivity for infectious pathogens. In addition, microbial EV-based vaccines show promise in enhancing immune protection while reducing side effects^[231]^. Furthermore, utilizing these vesicles as nanocarriers for delivering antimicrobial drugs or genetic therapeutics provides new strategies for anti-infective treatments.

## APPLICATIONS OF EVs IN BIOMEDICAL FIELDS

EVs carry a rich cargo of biomolecules that reflect the physiological or pathological status of their parent cells, offering significant potential for both diagnosis and therapy and serving as valuable theranostic resources. Driven by advances in isolation, characterization, and engineering strategies, EV-based theranostic research is accelerating and is expected to provide essential support for precision medicine^[232]^.

### Engineering approaches of EVs

**Figure 7:**
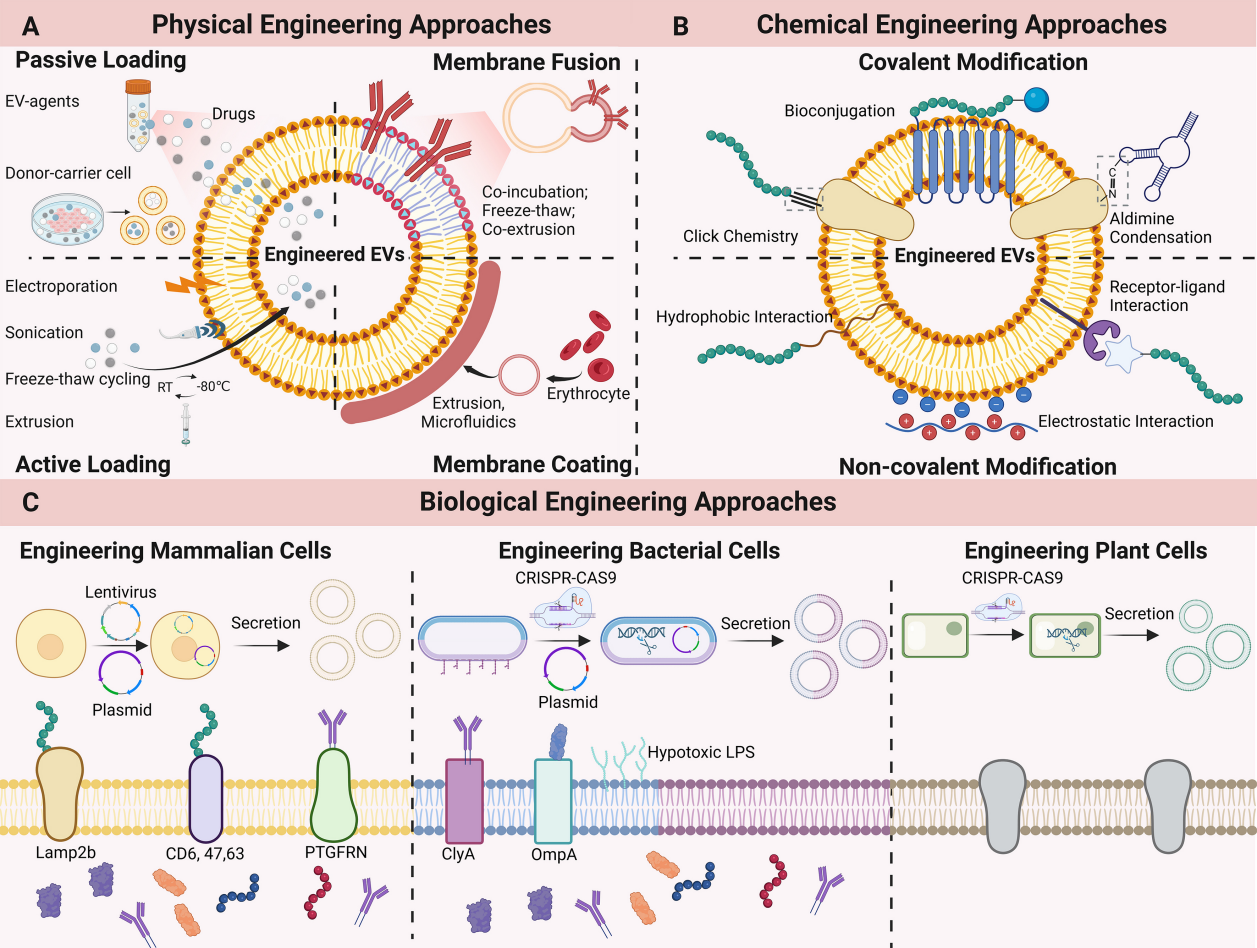
Representative Methods of EV Engineering. (A) Physical engineering of EVs. Physical methods enable therapeutic agent loading and membrane functionalization. Passive loading is achieved through co-incubation, while active loading employs techniques such as electroporation, sonication, extrusion, and freeze-thaw cycling. Membrane modification can be accomplished via membrane fusion (utilizing co-incubation, freeze-thaw, or extrusion) or membrane coating (via extrusion or microfluidics); (B) Chemical engineering of EVs. Chemical modification strategies are divided into covalent and non-covalent approaches. Covalent methods include click chemistry, bioconjugation, and aldehyde-amine condensation. Non-covalent modifications leverage hydrophobic interactions, electrostatic interactions, or receptor-ligand binding; (C) Biological engineering of EVs. In mammalian cells, plasmid transfection or lentiviral infection enables the expression of fusion proteins, wherein targeting peptides or proteins are displayed on the EV membrane. Similarly, bacterial cells can be engineered with plasmids to overexpress surface fusion proteins. Additionally, the CRISPR-Cas9 system can be utilized to knock out genes associated with toxicity or immunogenicity, or to modify plant-derived EVs. EVs: Extracellular vesicles; RT: room temperature; LPS: lipopolysaccharide; CRISPR-CAS9: clustered regularly interspaced short palindromic repeats-CRISPR associated protein 9; Lamp2b: lysosome-associated membrane glycoprotein 2b; PTGFRN: prostaglandin F2 receptor negative regulator; ClyA: cytolysin A; OmpA: outer membrane protein A.

Although EVs show promising potential as therapeutic vehicles, their inherent targeting ability is limited, which impedes their broader utility. To achieve optimal therapeutic efficiency, a variety of engineering approaches, mainly including physical, chemical and biological approaches, have been developed to endow EVs with auxiliary functionalities such as enhanced targeting, penetrating, and multiple bioeffects^[233,234]^ [Figure 7].

#### Physical engineering approaches

Physical engineering approaches are commonly utilized to encapsulate therapeutic agents such as small-molecule drugs, nucleic acids, peptides, and proteins into EVs. These approaches may be categorized into passive loading and active loading. Passive loading typically includes EV-agent co-incubation of EVs with therapeutic agents or donor cells with carrier molecules, whereas active loading involves techniques such as electroporation, sonication, extrusion, freeze-thaw cycling, and other methods. Beyond drug encapsulation, physical engineering approaches are also exploited to functionalize the EV membrane. Strategies such as membrane fusion and membrane coating can enhance the targetability of EVs and reduce their immunogenicity, thereby facilitating their clinical application^[235]^.

#### Passive loading

The hydrophobic nature of the EV lipid bilayer allows hydrophobic drugs (e.g., curcumin, paclitaxel, and azithromycin) to passively diffuse into the vesicles along a concentration gradient^[236]^. In addition, miRNAs can also be loaded into EVs through passive loading, enabling efficient delivery and targeted therapy^[237]^.

Donor-carrier cell co-incubation is another passive loading strategy. For instance, apoptotic cells can efficiently encapsulate chemotherapeutic drugs or nanoparticles into EVs^[238]^. In addition, autologous malignant cells isolated from malignant pleural effusions can be incubated with methotrexate (MTX) to produce EVs that retain tumor-targeting and immunostimulatory capacities while delivering MTX for synergistic therapy^[239]^.

#### Active loading

Electroporation is currently the most widely used active loading method that applies short, high-voltage pulses to transiently permeabilize EV membranes, enabling cargo entry. Many small molecules (e.g., chemotherapeutic drugs, miRNA, siRNA, and luminescent substances)^[183,240]^ and macromolecules (e.g., DNA^[241]^ and proteins^[242]^ can be loaded into EVs via electroporation. However, electroporation may affect EV stability. To address this, microfluidic-assisted electroporation has been developed, which reduces voltage requirements and minimizes membrane damage^[243]^.

Sonication is another common membrane penetration strategy. Ultrasound-induced mechanical shear disrupts EV membranes, thereby enhancing cargo diffusion. This method is commonly used to load chemotherapeutic agents into EVs, and shows higher loading efficiency than electroporation^[244]^. However, several drawbacks, including drug adhesion to EV surfaces and loss of EV structural integrity, limit its widespread application.

Extrusion involves pressing a mixture of EVs and liposomes (Doxil) through polycarbonate membranes (e.g., with pore sizes of 400, 200, or 100 nm) under physical pressure to form hybrid vesicles with more controlled and uniform sizes compared to incubation-based methods^[245]^. Although extrusion demonstrates high fusion efficiency, the shear stress generated during the process may disrupt EV membrane integrity^[246]^.

Freeze-thaw cyclizing is a relatively simple physical process that disrupts membranes through ice crystal formation. This method removes water molecules from the hydrophilic surfaces of lipid bilayer membranes, leading to significant changes in membrane structure and function^[247]^. However, this method can induce EV aggregation, leading to increased particle size.

Saponin treatment represents another strategy to alter EV membrane permeability. By selectively removing cholesterol from the lipid bilayer membranes, saponin induces pore formation, thereby facilitating the loading of bioactive molecules such as lipids^[248]^. However, the potential cytotoxicity of saponin hinders its widespread applications, necessitating careful control of its concentration and thorough removal of residuals after loading.

Different loading methods result in varying efficiencies for the same cargo. Haney *et al.* evaluated the loading, storage, and release of peroxidase encapsulated into EVs using several methods, including incubation, ultrasound-induced sonication, extrusion, freeze-thaw cycling, and saponin permeabilization^[249]^. Sonication and extrusion showed higher efficiency than simple incubation, whereas freeze-thaw cycling and saponin permeation offered moderate efficiency. The hydrophilicity of the loaded therapeutic agents is a key factor affecting the loading efficiency. Notably, sonication, extrusion, and freeze-thaw cycling tended to increase EV size, whereas co-incubation at room temperature did not significantly alter particle size. Overall, both the passive and active loading strategies described above have their own advantages and limitations. Therefore, the optimal technique should be selected based on the specific cargo and intended application to achieve efficient EV loading.

#### Chemical engineering approaches

Chemical engineering approaches are commonly exploited to display functional molecules or ligands on the surface of EVs. Based on the functionalization strategy, these approaches can be classified into two categories: covalent modifications, including click chemistry, bioconjugation and aldehyde amine condensation, and non-covalent modifications such as hydrophobic insertion, multivalent electrostatic interaction, and receptor-ligand binding.

#### Covalent modification

Click chemistry, bioconjugation, and aldehyde-amine condensation are three common modification strategies for covalently linking exogenous functional groups and bioactive molecules to EV membranes. Click chemistry is a copper-catalyzed azide-alkyne cycloaddition process^[250]^. Typically, an alkyne group is first attached to EVs via EDC [N-(3-Dimethylaminopropyl)-N’-ethylcarbodiimide hydrochloride]-NHS (N-hydroxysuccinimide)-mediated condensation. Subsequently, under copper catalysis, the alkyne moiety reacts with azide-bearing functional molecules to form a stable covalent bond. Smyth *et al*. utilized the method to prepare Fluor-545-modified EVs without changing the size and function of EVs^[251]^. To circumvent potential copper-induced cytotoxicity, copper-free click chemistry has also been developed^[252]^.

Bioconjugation enables the display of functional macromolecules on EV surfaces. For example, the peptide CP05 can bind specifically to the second extracellular loop of the EV marker CD63^[253]^. Gao *et al*. separately conjugated the muscle-targeting peptide M12 and a Duchenne muscular dystrophy treatment drug PMO to CP05. Incubating EVs with the resulting CP05-M12 and CP05-PMO conjugates yielded dually functionalized EVs with enhanced targeting and therapeutic efficacy^[254]^. Theoretically, CP05 can serve as a universal anchor for diverse cargoes, offering broad potential in EV isolation, imaging, and targeted therapy^[255]^.

Aldehyde-amine condensation reaction offers a direct means to modify EVs with functional nucleic acid aptamers^[256]^. Due to the high affinity and specificity of aptamers, aptamer-conjugated EVs represent a promising platform for precision medicine.

#### Non-covalent modification

Hydrophobic insertion, electrostatic interactions, and receptor-ligand binding are the three most used non-covalent methods for EV surface engineering. EV membranes share a similar amphiphilic lipid bilayer structure with cell membranes, composed mainly of phospholipids, cholesterol, and glycolipids. The synthesized amphiphiles have hydrophilic heads and hydrophobic tails. Therefore, amphiphiles attached to different functional groups and materials can efficiently embed themselves into the cell membranes and EVs through hydrophobic interactions. In hydrophobic insertion, bioactive molecules are typically conjugated to hydrophobic anchors such as DSPE-PEG, palmitic acid, or cholesterol. The strong hydrophobic nature of these anchors facilitates their spontaneous integration into the EV lipid bilayer^[257]^.

Electrostatic interaction involves the mutual attraction of negative and positive charges. Since most EV surfaces are negatively charged, cationic polymers can readily associate with them through electrostatic interactions. For instance, simple mixing of EVs with cationized pullulan - a polysaccharide targeting hepatocyte asialoglycoprotein receptors - resulted in pullulan-modified EVs that showed enhanced liver accumulation and efficacy in treating liver injury^[258]^.

Receptor-ligand interaction generally involves modifying EVs with a protein receptor or ligand to enable specific binding to complementary molecules. For instance, Yang *et al.* used clusters of transferrin, which naturally associate with blood-borne EVs, to couple superparamagnetic nanoparticles for isolation purposes^[259]^. Alternatively, non-natural receptor moieties can be engineered onto EVs. Weyant *et al.* developed a generalized strategy that fuses the biotin-binding proteins with outer membrane scaffolding proteins of bacterial OMVs through genetic engineering^[231]^.

#### Biological engineering approaches

Biological engineering is widely used to functionalize EV membranes with fusion proteins or targeting peptides. Owing to the rapid development of genetic engineering and synthetic biology techniques, gene sequences encoding desired proteins or peptides can be designed and fused with selected membrane protein genes. These rationally synthesized gene constructs can be transfected into mammalian, bacterial, or plant cells, leading to the production of EVs that display the functional proteins or peptides on their surface.

Beyond membrane engineering, biological engineering approaches are also utilized to overexpress bioactive proteins, peptides, or miRNAs inside parent cells, thereby enriching the resulting EVs with bioactive cargo^[260]^. Furthermore, gene knockout techniques can be applied to eliminate specific genes, generating EVs with reduced toxicity or immunogenicity.

Targeted modification of EVs can be achieved by genetically engineering transmembrane proteins in parent cells. EV transmembrane proteins can be categorized into two categories: nonspecific proteins and receptor membrane proteins. Various nonspecific proteins have been identified, including Lamp2b and CD63, which are naturally enriched on EV membranes from parental cells^[261]^. Nonetheless, these conventional scaffolds face limitations in the expression level. To circumvent the expression level limitation, researchers from Codiak BioSciences identified PTGFRN (prostaglandin F2 receptor negative regulator) as the naturally highly abundant surface protein of EVs, enabling its high-density surface display with over 30-fold greater efficiency^[262]^. The PTGFRN-based EV bioengineering technique, the engEx^TM^ platform, facilitates the development of therapeutic candidates such as exoIL-12, the world’s first entry of engineered exosome into Phase I clinical trial^[263]^. Besides, clustered regularly interspaced short palindromic repeats-CRISPR associated protein 9 (CRISPR-Cas9)-based knockout systems are available to remove specific effectors such as *msbA*, *lpxL1*, or *lpxM* in Gram-negative strains to produce OMVs with attenuated virulence. Overall, engineering of parental strains can generate EVs with low toxicity and immunogenicity, along with targeting and therapeutic capabilities, thereby improving therapeutic efficiency. Moreover, CRISPR-Cas9-based plant genome editing has been widely reported, offering a promising approach to produce engineered PDEs^[264,265]^. However, the limited availability of genetically engineered plants models, plant cell wall barriers, generally low editing efficiencies, and insufficient functional annotation of plant genes make these approaches challenging at present^[266]^.

### Application of EVs as biomarkers in disease diagnosis

EVs have garnered significant interest as promising biomarkers for liquid biopsy due to their presence in diverse body fluids and their cargo of disease-relevant biomolecules^[230,267]^. Detailed characterization of the molecular cargo within EVs provides unique insights into cellular physiology and pathological states, thereby facilitating the early diagnosis of a wide range of diseases.

#### EV proteins

EV protein biomarkers primarily fall into two categories: those with aberrant expression levels and those with aberrant posttranslational modification (PTM) patterns, with most studies focusing on the former. Proteomic analyses have identified numerous EV proteins as promising cancer biomarkers across malignancies, including but not limited to prostate^[268]^, ovarian^[269]^, breast^[270]^, bladder^[271]^ and lung cancers^[272]^. Notably, certain protein biomarkers demonstrate enhanced diagnostic or prognostic value when analyzed within EVs compared to their dissociated forms, such as prostate-specific antigen (PSA) for prostate cancer^[273]^, developmental endothelial locus-1 protein for breast cancer^[274]^, Eph receptor A2 (EphA2) for pancreatic cancer, and PD-L1 for monitoring responses to anticancer immunotherapy^[123]^. Furthermore, specific tumor EV proteins, such as macrophage migration inhibitory factor and integrins (e.g., α6β4 and αvβ5), could be used to predict organ-specific metastasis^[275]^ [Table 3].

**Table 3:** Typical surface proteins from circulating EVs of cancer patients

**Protein (combination)**	**Diseases**	**Source**	**Reference**
HER2	Breast cancer	Plasma	[276]
EphA2	Pancreatic cancer	Plasma	[277]
CA-125/EpCAM/CD24	Ovarian cancer	Plasma	[278]
EpCAM/PD-L1	-	Plasma	[279]
EpCAM/ASGPR1/CD147	Hepatocellular carcinoma	Plasma	[280]
MCSP/MCAM/ErbB3/LNGFR	Melanoma	Plasma	[281]
EGFR/EpCAM/HER2/MUC1/GPC1/WNT2	Pancreatic cancer	Plasma	[282]

HER2: Human epidermal growth factor receptor 2; EphA2: Eph receptor A2; CA-125: cancer antigen 125; EpCAM: epithelial cell adhesion molecule; CD24: cluster of differentiation 24; PD-L1: programmed death-ligand 1; ASGPR1: asialoglycoprotein receptor 1; CD147: cluster of differentiation 147; MCSP: melanoma-associated chondroitin sulfate proteoglycan; MCAM: melanoma cell adhesion molecule; ErbB3: erb-b2 receptor tyrosine kinase 3; LNGFR: low-affinity nerve growth factor receptor; EGFR: epidermal growth factor receptor; MUC1: mucin 1; GPC1: glypican 1; WNT2: Wnt family member 2.

Beyond changes in expression levels, PTMs enhance the structural complexity and functional diversity of proteins via covalent modification by functional groups. Aberrant PTMs are frequently identified in disease-related EVs, such as glycosylation, acylation, and phosphorylation. Glycans attached to proteins with nitrogen or oxygen linkages are classified as N- or O-glycosylation. Glycosylation, which involves the attachment of glycans via nitrogen (N-glycosylation) or oxygen (O-glycosylation) linkages, participates throughout EV biogenesis^[283]^. The typical glycoforms include sialic acids (α-2,3 and α-2,6 type), mannose, complex N-glycans, heparan sulfate and polylactosamine. Glycoproteins occupy the major parts of glycoconjugates on EVs, and aberrant glycosylation has been regarded as a disease biomarker. For example, elevated sialic acid levels on EV proteins promote immune inhibition in various cancers via the interaction between sialic acids and receptors (Siglec) on immune cells^[284]^. Similarly, glycosylated CD133 ascites-derived exosomes emerge as a potential prognostic biomarker for pancreatic cancer^[285]^. Other glycosylation types, such as mucin 1 (MUC1) with aberrant O-glycoforms and terminal fucosylation on haptoglobin, have been reported as potential cancer biomarkers or neoepitopes^[286,287]^.

Protein acylation is another frequent and essential type of PTMs, including acetylation, crotonylation, lactylation, palmitoylation, myristoylation, succinylation, β-hydroxybutyrylation, and malonylation^[288]^. Minic *et al*. identified upregulated acetylation and specific acetylation patterns in breast cancer-derived sEVs, and revealed that acetylated glycolytic metabolic enzymes in sEVs could be potential biomarkers for the early diagnosis of breast cancer^[289]^. Palmitoylation is widely reported in the exosome production including protein sorting and membrane organization^[290]^. Mariscal *et al*. identified palmitoyl-protein ABCC4 (adenosine triphosphate-binding cassette sub-family C member 4), human six-transmembrane epithelial antigen of the prostate (STEAP)1, and STEAP2 in EVs as prostate cancer-specific biomarkers, and inhibition of palmitoylation decreased the sorting of these proteins into EVs^[291]^.

Phosphorylation is a major regulatory mechanism for protein functions. Advances in phosphoproteomic technologies have enabled extensive profiling of phosphoproteins in EVs. There is evidence that plasma-derived extracellular vesicles carry more than 100 phosphorylated proteins, whose abundances are generally higher in breast cancer patients than in healthy individuals. In addition, Soares Martins *et al*. identified 150 proteins with altered expression and/or phosphorylation patterns in blood-derived EVs from Alzheimer’s Disease (AD) patients using a high-density phosphoproteome microarray^[292]^.

#### EV RNAs

Accumulating evidence indicates that a spectrum of RNA species, including mRNAs, miRNAs, lncRNAs, tsRNAs, and circRNAs, are selectively enriched within EVs. Among these, EV-derived miRNAs have been extensively studied due to their relative abundance and stability, with several EV miRNA-based assays already translated to clinical use. In 2015, Li *et al*. reported, for the first time, that circRNAs are enriched in exosomes, and serum exosomal circRNAs were able to distinguish cancer patients from healthy controls, highlighting EV circRNAs as promising cancer biomarkers^[42]^. They subsequently developed exoRBase database (www.exoRBase.org) that contains more than 160,000 EV long RNAs (termed exLRs), including mRNAs, lncRNAs, and circRNAs, derived from diverse human body fluids^[44-46]^. Importantly, about one-third of these EV RNAs reflect their tissue or cell origin, establishing a foundation for EV long RNA biomarker research^[293]^. In addition, tsRNAs in EVs also appear as promising biomarkers for various diseases^[294,295]^.

#### EV DNA

In 2014, Thakur revealed, for the first time, the presence of dsDNA in tumor-derived exosomes (exoDNA) that represents the whole genomic DNA and mutational status of parental cancer cells, proposing its translational potential as a circulating cancer biomarker^[125]^. Subsequent studies have reported the functions of EV DNA, such as activating immune response through horizontal gene transfer and dsDNA recognition pathways^[296,297]^. Interestingly, cells can remove harmful cytoplasmic DNA via exosome secretion to maintain cellular homeostasis^[298]^. Wortzel *et al*. described a unique structural configuration of EV DNA, and revealed that uniquely chromatinized EV DNA can regulate anti-tumor immunity^[299]^. These functional insights further support the biomarker potential of EV DNA.

However, a recent publication reported that sEVs are not primary vehicles of active DNA release and proposes an alternative model involving an autophagy- and MVB-dependent, but exosome-independent, mechanism for extracellular DNA secretion^[129]^. Given the disparate DNA detection methodologies employed across these studies, the presence and biological relevance of genomic DNA within EVs remains ambiguous. Nonetheless, research on EV DNA has substantially advanced the field^[300]^.

#### EV metabolomics

Metabolites, as downstream products of biochemical reaction networks, provide phenotypic information that more directly reflects an individual’s physiological and pathological state than genomic or proteomic data, offering significant potential for assessing disease status. Nishida-Aoki *et al*. revealed a substantial elevation of exosomal diacylglycerol in highly metastatic triple-negative breast cancer cells compared with those with lower metastatic potential, suggesting its potential as a biomarker for breast cancer progression^[301]^. Dorado *et al*. determined that the lipid composition of plasma EVs could efficiently differentiate breast cancer from healthy control with an accuracy of 93.1%^[302]^. Skotland *et al*. reported that a specific combination of two phosphatidylserine molecules and one lactosylceramide in urinary exosomes could accurately discriminate prostate cancer^[303]^. Notably, Chen *et al*. constructed a biomarker panel based on urinary exosomal metabolic features, which accurately discriminates early gastric cancer patients from healthy controls^[304]^. In addition, Krokidis *et al*. identified plasma EV lipidomic profiling as a powerful biomarker discovery tool in AD and related neurodegenerative conditions^[305]^. Collectively, these studies highlight the substantial potential of EV metabolites as circulating biomarkers.

### Applications of EVs in disease treatment

#### Applications of EVs in cancer therapy

The incorporation of EVs in cancer therapy stands as an emerging and highly promising field. Current approaches to tumor treatment are diverse, including surgical resection, minimally invasive interventional therapy, chemotherapy, radiotherapy, targeted therapy, immunotherapy, and physical therapy^[306]^. In clinical settings, patients with early-stage tumors show favorable prognoses after curative surgical excision, whereas the majority of advanced cancer patients require multifaceted, integrated treatment strategies to extend their survival and improve their quality of life^[307]^. In this section, we will review the research progress of EVs in cancer therapy.

#### Applications of EVs in chemotherapy

Chemotherapy holds a cornerstone in cancer therapy. However, toxic side effects and drug resistance significantly limit its clinical effectiveness. Novel liposome-based chemotherapeutic drugs, such as doxorubicin (Dox) liposomes (Doxil), have gained extensive clinical application, enhancing the efficacy and safety of chemotherapy^[308]^. Similarly, EVs, as biologically derived lipid nanovesicles, exhibit great potential as effective carriers for chemotherapeutic drug delivery. Analogous to Doxil, Dox-loaded exosomes (Exo-Dox) demonstrated markedly enhanced cellular uptake and anti-tumor efficacy, along with reduced cardiotoxicity^[309]^. Li *et al*. decorated milk exosomes with hyaluronan, a CD44-specific ligand, to specifically deliver Dox into CD44-overexpressing tumor cells^[310]^. In addition, they also used this engineered milk exosome system to deliver miR-204-5p, a pan-cancer tumor suppressor, into CD44-expressing cancer cells, efficiently inducing cancer cell death^[311]^. Beyond sharing the characteristics of lipid-based vesicles, EVs exhibit a unique capacity to traverse the blood-brain barrier (BBB). Recognizing this potential, Zhu *et al*. developed dual-targeted, functionalized sEVs modified with Angiopep-2 and Transactivator of Transcription (TAT) peptides to deliver chemotherapeutic agents. This system could traverse the BBB, efficiently inhibit glioma growth and reduce chemotherapy-related toxic side effects^[312]^.

EVs also show potential application to reverse chemoresistance. For example, miR-21, an oncogenic miRNA, has been shown to foster chemoresistance in multiple cancer types by suppressing targets such as phosphatase and tensin homolog (PTEN), human mutS homolog 2 (hMSH2) and programmed cell death 4 (PDCD4)^[313]^. Liang *et al*. harnessed sEVs to co-deliver miR-21 inhibitors and 5-fluorouracil (5-FU) to tumor cells, overcoming chemoresistance and enhancing treatment effects^[314]^. Similarly, cell-derived exosomes or milk exosomes have been used to deliver miR-204-5p, inhibiting drug resistance and tumor growth^[311,315]^. These studies underscore the potential of EV-mediated drug delivery in overcoming chemoresistance and enhancing treatment efficacy.

#### Applications of EVs in targeted therapy and immunotherapy

In recent years, targeted therapy has been extensively applied to cancer patients with specific molecular features. By precisely targeting molecules on tumor cells or immune cells, these therapies can effectively kill cancer cells while minimizing harm to normal cells^[316]^. EVs have attracted considerable attention as delivery vehicles or therapeutic agents for both targeted therapy and immunotherapy^[317]^.

Nucleic acid-based gene therapy is a crucial component of cancer-targeted therapy but faces challenges such as poor membrane permeability and susceptibility to enzymatic degradation. A robust delivery system is essential to protect nucleic acids and ensure their targeted delivery. EVs meet these demands with their excellent loading capacity, high stability, and biocompatibility^[318]^. Among emerging technologies, the CRISPR-Cas9 system stands out for its potential in gene-targeted treatments^[319]^, and EVs serve as promising vectors for this system^[320]^. In 2017, Kim *et al*. reported, for the first time, that tumor-derived EVs serve as a valuable delivery platform of CRISPR-Cas9 for cancer gene editing^[242]^. Wan *et al*. successfully integrated Cas9/single-guide RNA (sgRNA) ribonucleoprotein (Cas9 RNP) into exosomes secreted by hepatic stellate cells (HSCs), creating a precise and tissue-specific gene therapy platform for liver diseases^[321]^. Furthermore, the remarkable capability of EVs to encapsulate and deliver nucleic acids has positioned them as crucial tools for advancing gene-targeted tumor therapy, enabling highly effective and precise treatments^[322]^.

Given that RNA is prone to degradation and has difficulty crossing cell membranes to enter cells, EVs, as natural lipid vesicles, emerge as promising delivery carriers that enhance RNA stability and targeting accuracy^[323]^. In numerous preclinical studies, EVs have demonstrated significant potential as delivery tools for nucleic acid drugs, including antisense oligonucleotides (ASOs), siRNA, miRNAs, mRNAs, circRNAs, and lncRNAs^[324]^. Codiak Biosciences, a pioneering company in the clinical translation of exosomes, utilized ASO-loaded EVs to knock down STAT6 (signal transducer and activator of transcription 6) in tumor-associated macrophages (TAMs), thereby remodeling TME and inhibiting tumor growth^[325]^. Chen *et al*. developed a dual-targeted milk exosome system to deliver PD-L1 siRNA (siPD-L1) into TAMs, efficiently reprogramming TAMs and TME^[326]^. Zhao *et al*. developed an exosome membrane-coated biomimetic nanoparticle system (CBSA/siS100A4@Exosome) to improve siRNA delivery to the lung pre-metastatic niche of breast cancer, achieving excellent gene knockdown and lung metastases inhibition^[327]^.

In addition to these small nucleic acids, mRNAs can also be efficiently encapsulated and delivered by EVs. In 2020, Yang *et al*. developed a method to efficiently encapsulate therapeutic mRNA into exosomes on a large scale. In a PTEN-deficient glioma mouse model, these mRNA-loaded exosomes restored anti-tumor defenses, suppressed tumor growth, and improved survival outcomes^[328]^. EV-based nucleic acid delivery enables personalized therapy by selecting optimal nucleic acid types for individual patients, paving the way for precise and effective clinical interventions.

Cancer immunotherapy, a specialized subset of targeted therapy, focuses on reversing immunosuppressive TME. Its central principle is to reinvigorate and enhance the body’s anti-tumor immune response^[329]^. EV-based immunotherapy is among the most promising anti-tumor strategies currently available^[330]^. In addition to the EV-delivered RNA drugs mentioned above that regulate anti-cancer immune response^[326]^, engineered EVs from tumor cells or immune cells also show potential as immune mediators. For example, engineered tumor-derived exosomes (TEXs) can serve as an attractive cell-free tumor vaccine platform^[331]^. Liang *et al*. have constructed a biologically self-assembled tumor nanovaccine platform with TEXs as an efficient drug delivery system for tumor antigens^[332]^. However, TEXs may contain tumor-promoting molecules, which limits their clinical application.

Apart from TEXs, DC-derived exosomes (DEXs) have also garnered significant attention as tumor vaccines. potentially surpassing DC-based vaccines in evading tumor cell-mediated immune suppression^[332]^. A notable example is the “trigger” DEX vaccine (DEXP&A2&N) developed by Zuo *et al*, which effectively triggers tumor-specific immune responses against hepatocellular carcinoma^[333]^. Nevertheless, obtaining sufficient immune cells and their EVs remains challenging. Furthermore, both TEXs and DEXs exhibit limited efficacy in activating anti-tumor immune responses. Consequently, combining these EVs with other treatment modalities is often necessary to achieve optimal therapeutic outcomes^[334]^.

Immunologically engineered EVs, modified with antibodies targeting cancer-promoting molecules such as CD3, human epidermal growth factor receptor 2 (HER2), PD-1, and PD-L1, could be utilized to modulate anti-cancer immune response^[330]^. In addition, Chimeric antigen receptor (CAR) T cell-derived EVs also show potential for anti-cancer therapy^[335]^.

#### Applications of EVs in physical therapy

In addition to conventional clinical modalities for cancer treatment, innovative therapeutic strategies leveraging physical stimuli such as light, sound, and magnetism are increasingly gaining widespread clinical acceptance. Treatments based on light include photothermal therapy and photodynamic therapy (PDT), among others. PDT has been approved by the FDA for clinical application^[336]^. Similarly, most photosensitizers are also sonosensitizers, enabling photoacoustic imaging and sonodynamic therapy (SDT) under ultrasonic stimulation^[337]^. A key advantage of SDT is the deeper tissue penetration of ultrasound compared to light, making it suitable for treating tumors located in deeper regions^[337]^. Magnetic field-based therapies typically involve magnetic hyperthermia, which relies on appropriate magnetic fields and magnetic materials such as iron oxide nanoparticles, particularly superparamagnetic iron oxide nanoparticles (SPIONs). Similar to sound, magnetic fields overcome penetration limitations, and SPIONs can also enhance MRI^[338]^.

A long-standing challenge in this field is improving the delivery efficiency of photosensitizers, sonosensitizers, and iron oxide nanoparticles. To address this, Cheng *et al*. engineered sEVs with a chimeric peptide for dual targeting of the cytoplasmic membrane and nucleus, loading them with photosensitizers to achieve more precise strikes against tumor cells while reducing drug toxicity and side effects^[339]^. Liu *et al*. designed a functionalized smart nano-sonosensitizer by loading sonosensitizers into engineering exosomes. This approach not only enhances the efficiency of drug delivery but also enables ultrasound-controlled drug release, thereby augmenting the anti-tumor efficacy of SDT^[340]^. Through innovative EV engineering combined with physical therapy^[341]^, researchers have significantly bolstered the efficacy of tumor eradication while maintaining rigorous biosafety standards.

#### Applications of EVs in comprehensive multimodal therapy

Monotherapy often shows limited efficacy in tumor treatment, necessitating combination therapy as a key strategy to improve therapeutic outcomes. Due to their excellent biocompatibility, tumor targeting, long circulation, immune-modulatory properties, and ease of modification, EVs have emerged as versatile platforms for comprehensive multimodal therapy, including phototherapy, chemotherapy, gene therapy, and immunotherapy^[252]^. They can co-deliver chemotherapeutic agents, genetic materials, immune modulators, and phototherapeutic compounds, offering a synergistic multimodal approach to combat tumor heterogeneity and resistance.

In a pioneering study, Guo *et al*. developed a novel therapeutic platform using hollow manganese dioxide (MnO_2_) particles loaded with the photosensitizer indocyanine green (ICG) and camouflaged by PD-L1 antibody-modified exosomes^[342]^. This platform combines PDT with immunotherapy, simultaneously targeting both the physical and immunological dimensions of tumors. In another study^[343]^, a cutting-edge nano-polymer (Ir-Fe NP) was developed by synthesizing an Ir(III) photosensitizer with Fe(III) and encapsulating it within melanoma-derived exosomes. This combined photodynamic and chemodynamic approach offers a highly efficient and potent strategy for melanoma treatment. Besides, Cheng *et al*. developed a hybrid nanovesicle by fusing genetically engineered exosomes with drug-loaded temperature-sensitive Doxil, establishing a promising nanomedicine platform for efficient and safe photothermal-immunotherapy combination therapy^[344]^.

Chemotherapy can activate anti-tumor immunity by inducing tumor cell death and releasing tumor antigens^[345]^. This effect often serves as a precursor to or is synergistically combined with immunotherapy, enhancing the overall treatment effectiveness by enabling the immune system to recognize and eliminate residual tumor cells. For example, a hybrid exosome system was developed by fusing M1-like macrophage-derived exosomes with genetically engineered TEXs carrying CD47^[346]^. This complex was loaded with the DNA-damaging agent SN38 and the stimulator of interferon genes (*STING*)-agonist MnO-_2_, resulting in multifunctional hybrid exosomes that exert excellent anti-cancer effects via multiple mechanisms. Moreover, Shan *et al*. developed a functionalized macrophage-derived exosomal delivery system capable of crossing the BBB, co-delivering the small molecule drug panobinostat and PPM1D (protein phosphatase, magnesium-dependent 1, delta)-siRNA for combined chemo-gene therapy of PPM1D-mutant gliomas^[347]^. Other researchers have also developed multifaceted therapeutic platforms that integrate EVs with synthetic components to facilitate tumor-targeted chemical, genetic, and photothermal therapy^[348]^.

#### Applications of EVs in CVDs

CVDs represent the leading cause of death worldwide, accounting for nearly one-third of all global deaths^[349]^. Despite advances in therapeutic strategies, the prevention and treatment of CVDs remain a major challenge. Emerging evidence indicates that EVs are involved in the pathological progression of CVDs, holding promising potential for monitoring and treating these diseases^[350]^.

#### Applications of EVs in atherosclerosis

Atherosclerosis is a major CVD driven by chronic inflammation and lipid deposition within arterial plaques. EVs have been identified in plaques and play a crucial role in the progression of atherosclerosis^[351]^. Given that the activation of ECs is a key step in the intimal lesion formation of atherosclerosis, endothelial progenitor cells (EPC)-derived EVs have been shown to regulate the progression of atherosclerosis. Li *et al*. reported that EPC-EVs transfer miR-199a-3p to inhibit specificity protein 1 (Sp1), suppressing ferroptosis in ECs and delaying atherosclerosis^[352]^. Macrophages also play a vital role in atherosclerosis. EVs derived from anti-inflammatory M2 macrophages have been used to reduce inflammation and hematopoiesis in atherosclerotic plaques^[353]^. In a recent study, Xie *et al*. demonstrated that M2 macrophage-derived EVs fused with platelet membranes show enhanced plaque targeting and alleviate atherosclerosis development^[354]^. These findings highlight that EVs are promising therapeutic tools for atherosclerosis, warranting further investigation.

#### Applications of EVs in myocardial ischemia and injury

Myocardial ischemia, injury, and infarction represent distinct types of myocardial tissue damage caused by an imbalance between myocardial blood supply and oxygen demand. Recent studies have reported increased numbers of platelet-, EC-, and leukocyte-derived EVs in myocardial infarction (MI)^[355]^. Interestingly, these EVs have been shown to protect the myocardium from ischemia-reperfusion injury^[356]^. For example, EVs purified from serum-converted human platelet lysates provide robust protection following cardiac ischemia/reperfusion injury^[357]^. Similarly, EVs derived from Krüppel-like Factor 2 (KLF2)-overexpressing ECs attenuate myocardial ischemia/reperfusion injury by shuttling miR-24-3p that inhibits lymphocyte antigen 6C (Ly6C)^high^ monocyte recruitment^[358]^. Apart from these endogenous EVs, EVs derived exogenously from pluripotent stem cells also exert cardioprotective effects. MSC-EVs protect myocardial ischemia-reperfusion injury in mice by transporting miR-182 and thereby modulating macrophage polarization^[359]^. Hypoxia-induced MSC-EVs promote ischemic cardiac repair through miR-125b-mediated amelioration of cardiomyocyte apoptosis and prevention of cardiomyocyte death in MI^[360]^. Furthermore, sEVs derived from M2 macrophages inhibit pro-inflammatory C-C motif chemokine receptor 2 (CCR2)^+^ macrophage subpopulations to promote cardiac repair in MI^[361]^. In summary, EVs hold significant potential for mitigating myocardial ischemia-reperfusion injury by promoting myocardial repair through multiple mechanisms. These multifunctional properties position EVs as promising therapeutic candidates for the treatment of ischemic heart disease.

#### Applications of EVs in cardiac hypertrophy and heart failure

EVs have been documented to mediate crosstalk between cardiomyocytes and fibroblasts in the context of cardiac hypertrophy and heart failure. Under pathological conditions of these diseases, EVs derived from fibroblasts transfer miR-21 and miR-27a into cardiomyocytes, contributing to cardiac hypertrophy^[362]^. Similarly, macrophage-derived EVs transport miR-155 into fibroblasts, exacerbating cardiac fibrosis^[363]^. Conversely, stem cell-derived EVs carrying miRNAs such as miR-185-5p and miR-143/145 cluster can reduce the expression of genes associated with cardiac hypertrophy, offering protective effects^[202]^. Current research in this area is largely focused on profiling miRNA changes in circulating EVs as potential biomarkers for myocardial hypertrophy and heart failure. Studies exploring the therapeutic application of EVs and their miRNA cargo for treating these conditions are still in the early stages, highlighting a promising avenue for future investigation.

#### Applications of EVs in metabolic diseases

Applications of EVs in obesity Obesity is a serious worldwide epidemic characterized by long-term imbalance between caloric intake and energy expenditure. Visceral adipose tissue (VAT) regulates immunometabolic processes and systemic homeostasis through cellular signaling and cytokine release. Given the interplay between lipid metabolism and EVs, and the fact that their imbalance is closely related to obesity, EV-based therapies hold significant potential as a treatment for obesity^[364]^.

EVs play a dual role in adipocyte differentiation. They can actively promote terminal adipocyte differentiation via the MAPK-ERK signaling pathway. Conversely, Wang *et al*. found that tumor-derived EVs negatively affected adipose-derived MSCs (AD-MSCs) adipogenesis by activating TGF-β signaling pathway^[365]^. Additionally, miR-450a-5p in adipose tissue-derived exosomes (ATDEs) is upregulated during adipogenesis and promotes AD-MSCs adipogenesis by inhibiting WISP2 (WNT1-inducible signaling pathway protein 2)^[366]^. Dai *et al*. further demonstrated that ATDEs induce the expression of adipogenic marker genes in AD-MSCs and enhance the formation of lipid droplets^[367]^. Taken together, these findings highlight the crucial role of EVs in adipose differentiation, offering valuable therapeutic strategies for obesity.

EVs play a crucial role in regulating lipid metabolism and hold promise for obesity treatment. Zhao *et al*. reported that M2 macrophages treated with EVs derived from adipose-derived stem cells (ADSCs) express high levels of tyrosine hydroxylase, which in turn promotes ADSC proliferation and lactate production, thereby facilitating white adipose tissue (WAT) beiging and improving metabolic homeostasis^[368]^. Conversely, exosomal miR-155 from adipose tissue macrophage (ATM) is upregulated in high-fat diet mice and inhibits WAT beiging and thermogenesis, partly by targeting peroxisome proliferator-activated receptors (PPAR)γ^[369]^. Other obesity-related EV miRNAs, such as miR-122, miR-192 and miR-27a-3p, may serve as therapeutic targets for obesity^[364]^. In another study, melatonin treatment increased exosomal α-ketoglutarate levels and exosome release from adipose tissues, promoting anti-inflammatory ATM polarization^[370]^. TEXs also modulate lipid metabolism. For example, pancreatic cancer-derived exosomes carrying adrenomedullin trigger lipolysis, with similar effects observed with cancer-associated fibroblast-derived EVs^[371,372]^. Collectively, these findings underscore the pivotal role of EVs in lipid metabolism and their potential as therapeutic tools for obesity.

#### Applications of EVs in liver regeneration

The liver, a highly regenerative organ, relies on hepatocyte proliferation to maintain physiological homeostasis. Despite this capacity, end-stage liver disease remains a leading cause of death, with transplantation being the only curative option - limited by donor shortages. As an alternative, EVs have emerged as promising modulators of liver regeneration due to their cargo of growth factors, cytokines, and miRNAs that enhance hepatocyte proliferation and survival^[373,374]^. During liver injury, EVs mediate intercellular communication among hepatocytes, sinusoidal ECs, and immune cells to coordinate regenerative responses^[375]^. For instance, hepatocyte-derived exosomal miR-146a-5p induces HSC inactivation by promoting mesenchymal transition^[376]^. Conversely, mannan-binding lectin-associated serine protease-1 (MASP1) and the lncRNA cytoskeleton regulator RNA (CYTOR) in hepatocyte-derived EVs can activate HSCs and promote liver fibrosis^[377,378]^. Notably, HSC autophagy can suppress fibrosis by limiting fibrogenic EV release^[379]^. Exogenous EVs, particularly from stem cells, can deliver pro-regenerative and anti-inflammatory factors that enhance liver repair while avoiding risks such as tumorigenicity and immune rejection associated with cell therapy^[380]^.

Owing to their size and composition, EVs and other nanoparticles preferentially accumulate in the liver after intravenous injection. However, the primary liver cell type responsible for EV uptake remains debated. Hepatocytes, Kupffer cells, and ECs have all been shown to internalize EVs and contribute to regenerative signaling^[381,382]^. Notably, lipid nanoparticle (LNP)-mRNA delivery studies indicate hepatocytes as the primary target, followed by endothelial and Kupffer cells^[383]^, highlighting the importance of EV-cell interactions in liver repair.

#### Applications of EVs in diabetes

Accumulating evidence suggests that EVs, particularly exosomes, can serve as reliable, non-invasive biomarkers for early diabetes detection and disease progression monitoring^[384,385]^. Beyond diagnostic applications, EVs also play active roles in modulating metabolic pathways. Some miRNAs, proteins, and lipids encapsulated within EVs can influence key signaling cascades involved in glucose homeostasis, enhancing pancreatic β-cell functions and insulin sensitivity in peripheral tissues^[386]^. MSC-EVs have shown therapeutic promise for their capabilities in attenuating systemic inflammation, reducing oxidative stress, and promoting regeneration of pancreatic islets^[275,387]^. These regenerative and immunomodulatory effects make MSC-EVs attractive candidates for cell-free therapeutic strategies in both type 1 and type 2 diabetes. Taken together, EVs represent a versatile platform for next-generation diabetes therapeutics.

#### Applications of EVs in aging and NDs

EVs have emerged as key players in aging and rejuvenation^[388,389]^. Studies show that EVs from young plasma or cultured cells can rejuvenate aged tissues by enhancing mitochondrial function, reducing oxidative stress, and reversing cellular senescence^[390,391]^. MSC-EVs are particularly promising for combating aging-associated diseases by mitigating oxidative damage, promoting tissue repair, and extending health span in progeroid mice^[392,393]^. Conversely, senescent cells release significantly more EVs, which transmit paracrine senescence to neighboring cells, a process partially mediated by sEV IFITM3 (interferon-induced transmembrane protein 3)^[394]^. This functional duality underscores the importance of source selection in EV-based therapy. Future research should focus on optimizing therapeutic EVs from young or engineered cells while mitigating the adverse effects of senescence-associated EVs. Overall, EVs represent a compelling avenue for combating age-related decline and extending healthy lifespan.

NDs, characterized by progressive loss of neurons due to the accumulation of abnormal proteins or peptides in the central and peripheral nervous systems, account for millions of global deaths and disabilities annually^[395]^. Despite ongoing research, effective treatments remain limited, largely due to the BBB that restricts most therapeutic agents from entering the brain. EVs offer a promising solution due to their ability to cross the BBB and represent an ideal carrier for therapeutic molecules, enabling the delivery of repair and regulatory factors from stem cells to damaged neural tissue^[396]^.

#### EVs in AD

There are two main pathological mechanisms for AD: (1) Excessive cleavage of amyloid precursor protein (APP) by β-secretase rather than α-secretase, resulting in the accumulation of polymerized amyloid-β in the brain; and (2) Hyperphosphorylation of Tau proteins, forming neurofibrillary tangles that lead to neuronal dysfunction and cell death^[397]^. As EVs have been identified as carriers of pathological molecules during disease progression, pharmacological modulation of the release of EVs containing pathogenic cargos represents a common approach for ND therapy development^[398]^. For example, neuron-derived exosomes aid in forming non-toxic amyloid β (Aβ) protofibrils and enhance Aβ uptake and degradation by microglia. Their secretion is regulated by enzymes such as nSMase2 (neutral sphingomyelinase 2) and sphingomyelin synthase (SMS)2, positioning them as potential therapeutic targets for AD^[399]^.

Several studies demonstrate the therapeutic potential of engineered EVs in AD models. Liu *et al*. found that bone marrow MSCs (BMSCs)-derived exosomes alleviate cognitive decline in a constructed AD mouse model by improving brain-derived neurotrophic factor (BDNF)-related neuropathology^[400]^. Similarly, engineered MSC-EVs with high SHP2 (Src-homology 2 domain-containing phosphatase 2) expression significantly promote neuronal mitophagy, thereby reducing mitochondria damage-induced apoptosis and NLRP3 (NOD-, LRR- and pyrin domain-containing protein 3) inflammasome activation^[401]^. In addition, AD-MSC exosomes loaded with miR-122 show promising therapeutic potential in AD^[402]^. Yu *et al*. constructed engineered exosomes modified with rabies virus glycoprotein (RVG) peptide (RVG-EXO) and loaded with a neprilysin variant to target α7-nAChR in the brain’s hippocampus. RVG-EXO administration reduced pro-inflammatory markers (IL-1α, TNF-α, NF-ĸB) and elevated anti-inflammatory IL-10 in the brain, providing a novel therapeutic strategy for AD^[403]^.

#### EVs in Parkinson’s disease

The pathological features of Parkinson’s disease (PD) include the progressive degeneration of dopaminergic neurons in the substantia nigra, resulting in reduced dopamine levels and motor symptoms such as tremor and rigidity^[404]^. EVs not only participate in PD development and progression but also show potential as biomarkers and therapeutic tools^[405]^. In 2011, Alvarez-Erviti first demonstrated the therapeutic potential of exosomes for brain delivery by engineering immature DEXs expressing the fusion protein of Lamp2b-RVG. The endogenous engineered EVs were utilized to deliver siRNA into the brain to knock down β‑secretase 1 (BACE1, a therapeutic target in AD).

Liu *et al*. developed a core-shell hybrid nanosystem (REXO-C/ANP/S) coated with RVG-modified exosomes derived from immature DCs to deliver siRNA targeting α-synuclein (siSNCA), which can efficiently eliminate α-synuclein aggregates and reduce cytotoxicity in PD neurons^[406]^. Additionally, preventing dopaminergic neuron loss in the substantia nigra represents a promising therapeutic strategy for PD. Researchers employed umbilical cord blood mononuclear cell-derived sEVs to deliver miR-124-3p. These miR-124-3p-loaded sEVs successfully protected dopaminergic neurons from the 6-hydroxydopamine toxicity in a PD mouse model^[407]^.

Encapsulating therapeutic agents with low biocompatibility into EVs has emerged as a major research strategy for PD treatment. For instance, blood-derived exosomes loaded with dopamine significantly enhanced striatal and nigral dopamine distribution while showing increased therapeutic efficacy and reduced systemic toxicity compared to free dopamine in PD models^[408]^. Similarly, Luo *et al*. developed a milk exosome-based delivery system that efficiently delivers epicatechin gallate into SH-SY5Y cells and exhibits enhanced neuroprotective effects^[409]^. Stem cell-derived EVs also show therapeutic potential. EVs derived from human NSCs exhibit significant neuroprotective effects in PD models by reducing reactive oxygen species (ROS) and pro-inflammatory factors^[410]^.

#### Applications of EVs in trauma medicine

EVs in regeneration of the musculoskeletal system The musculoskeletal system, essential for movement and postural maintenance, is vulnerable to various injuries and degenerative conditions^[411]^. Although surgical options exist, outcomes are often suboptimal, highlighting the need for innovative regenerative strategies. EVs have gained prominence in regenerative medicine, demonstrating their potential to facilitate the healing of wounds, bones, cartilage, menisci, spinal cord, rotator cuffs, tendons, and muscle fibers**.** For example, Sahu *et al*. revealed that EVs derived from young animals rejuvenate aged skeletal muscle regeneration^[412]^. MSC-EVs appear to be a promising novel therapy for musculoskeletal regeneration. In addition, hybridized nanovesicles based on stem cell-derived exosomes and hydrogels have shown enhanced effects in musculoskeletal regeneration^[413]^. A recent review summarized the therapeutic potential of EVs in musculoskeletal regeneration^[414]^.

#### EVs in wound healing

Wound healing is a complex clinical challenge involving various injuries, including infectious, diabetic, and burn wounds. This intricate process requires a coordinated interplay of cellular elements, growth factors, extracellular matrix, nerve fibers, and blood vessels. MSCs are key players in skin regeneration and repair, influencing all phases of wound healing from inflammation to tissue reconstruction^[415]^. MSC-EVs replicate the biological effects of their parent MSCs and have demonstrated potential applications in wound repair^[416]^. For example, MSC-EVs can promote M2 macrophage polarization and enhance cutaneous wound healing^[417]^. Additionally, EVs from M2 macrophages can induce the transition from M1 to M2 phenotype, accelerating wound healing by enhancing angiogenesis, re-epithelialization, and collagen deposition^[418]^. In addition, engineered MSC-EVs or MSC-EV-based hybrid vesicles have been constructed to enhance the effects of MSC-EVs in wound healing^[419]^. Furthermore, EVs from other stem cell types, such as ADSCs, have also shown potential in wound healing^[420]^.

#### EVs in bone regeneration

Bone defects pose significant clinical challenges. BMSCs play a pivotal role in bone regeneration, and BMSC-derived EVs produce therapeutic effects comparable to those of BMSCs in stimulating bone formation^[421]^. EVs enhance osteogenesis by stimulating the differentiation of MSCs into osteoblasts and promoting the deposition of extracellular matrix. On the one hand, EVs are commonly used to modify scaffolds for the repair of bone defects. For example, Youseflee *et al*. developed a hydroxyapatite scaffold loaded with EVs derived from endometrial MSCs, exhibiting satisfactory osteogenic and angiogenic characteristics^[422]^. Wang *et al*. developed an acellular fish scale scaffold enriched with BMSC-EVs for treating calvarial defects, demonstrating that the scaffold significantly promotes bone regeneration by enhancing the osteogenic differentiation of BMSCs^[423]^. BMSC-EV-loaded hydrogels have also been used to repair calvarial defects, exhibiting excellent thermosensitive properties and further enhancing bone regeneration. Additionally, stem cell-derived EVs or bacterial EVs can be employed to deliver therapeutic agents, such as growth factors, directly targeting injury sites to enhance bone healing^[424,425]^.

In addition to bone defects and fractures, EVs have been studied for the treatment of femoral head necrosis. Chen *et al*. developed an extracellular matrix-mimicking hydrogel composed of methacryloylated type I collagen-loaded EVs, which promotes bone repair in glucocorticoid-induced osteonecrosis of the femoral head by enhancing macrophage M2 polarization, osteogenesis, and angiogenesis to^[426]^. Overall, EVs represent a promising therapeutic tool for bone defect repair, fracture repair, and femoral osteonecrosis by promoting osteogenic differentiation of BMSCs and stimulating neovascularization.

#### EVs in cartilage regeneration

Cartilage is a connective tissue composed of chondrocytes that generate a collagen-rich extracellular matrix abundant with proteoglycans and elastin. However, it has a limited capacity for self-repair due to its poor vascularization, sparse cellular content, and scarcity of progenitor cells. Cartilage defect is a common clinical challenge with limited treatment options. EVs derived from MSCs or ADSCs have garnered attention as a viable therapeutic for post-traumatic cartilage injury and osteoarthritis of the knee^[427]^. However, clinical translation has been hindered by challenges such as inconsistent dosing and rapid *in vivo* clearance. To address these limitations, scaffolds or hydrogels loaded with EVs have been recently investigated.

Li *et al*. designed a biomimetic double-network hydrogel scaffold via 3D printing using tissue-specific decellularized extracellular matrix and human adipose MSC-EVs. This scaffold significantly enhanced simultaneous regeneration of cartilage and subchondral bone in a rat model^[428]^. A recent systematic review summarized the effect of EVs-loaded scaffolds on post-traumatic cartilage injury and knee osteoarthritis^[429]^. Collectively, EVs represent a promising future treatment option for cartilage defects and osteoarthritis.

#### EVs in meniscus injury repair

Meniscal injuries, commonly caused by sports activities or age-related degeneration, often lead to impaired knee function and increase the risk of osteoarthritis. Current surgical treatments yield limited success, especially in the avascular zones of the meniscus. EVs, particularly MSC-EVs, have shown great potential in promoting meniscal regeneration through enhancing meniscal cell migration and ECM synthesis, as well as inhibiting inflammatory responses^[430]^. In addition to MSC-EVs, exosomes from skeletal stem cells also offer a promising therapeutic strategy for meniscal injury repair^[431]^. Recent research has increasingly focused on combining EVs with bioengineered scaffolds to enhance meniscus regeneration.

#### EVs in spinal cord injury repair

Spinal cord injuries (SCI) are one of the most debilitating musculoskeletal injuries, often leading to permanent loss of motor and sensory functions. The CNS has limited capacity for self-repair. EVs can effectively cross the BBB, making them ideal candidates for drug delivery. Stem cell-derived EVs have emerged as a novel therapeutic approach for SCI due to their neuroregenerative properties and ability to modulate the post-injury microenvironment^[432]^. Specifically, EVs derived from NSCs and MSCs carry neuroprotective molecules, including miRNAs, growth factors, and anti-apoptotic proteins that can promote axonal growth and prevent neuronal cell death^[433,434]^. In addition, autologous plasma-derived EVs have been utilized for targeted delivery of growth-facilitating peptides to promote recovery after SCI^[435]^. These findings highlight the dual potential of EVs as both direct therapeutic agents and targeted delivery systems in SCI treatment.

In summary, EVs represent a versatile therapeutic tool for trauma medicine. Their integration into biomaterials such as hydrogels and scaffolds further enhances their regenerative potential, offering new avenues for treating complex musculoskeletal injuries.

#### Applications of EVs in infectious diseases

Infectious diseases, caused directly or indirectly by microorganisms, pose a significant threat to global public health. Chemotherapy remains the most effective option for the clinical management of infected patients. However, the emergence of multidrug-resistant (MDR) and extensively drug-resistant (XDR) strains has introduced challenges, including complex drug regimens and prolonged treatment durations^[436]^. To address these issues, EV-based nanotechnologies have been designed and developed for the diagnosis and treatment of infectious diseases^[437]^.

#### EVs in viral infection

EVs are closely linked to viral infection and body immunity. Many EV-based strategies have been designed to resist viral infection, including HIV^[438]^, severe acute respiratory syndrome coronavirus 2 (SARS-CoV-2)^[439,440]^ and HBV^[441]^. For example, Li *et al*. reported biomimetic macrophage EV-like nanoparticles coated with an aggregation-induced emission (AIE) molecule (TBD@M NPs) for virus transmission blockage and targeted photothermal therapy in monkeypox^[442]^. This pioneering study demonstrates the potential of nanovesicles in monkeypox theranostics and may pave the way for the development of promising therapeutic strategies for monkeypox management in clinical trials. Engineered EVs modified with the receptor-specific ligands have been applied to deliver therapeutic drugs to infected tissues or organs. Cas9/guide RNA (gRNA)-loaded EVs decorated with vesicular stomatitis virus-glycoprotein (VSV-G) significantly enhanced the endosomal escape of Cas9 protein and increased its gene-editing activity in recipient cells, effectively reducing viral antigens and HBV covalently closed circular DNA (cccDNA) levels in the HBV-replicating and infected cells^[441]^. In addition, the innovative approach of incorporating virus-encoded envelope proteins into EVs carrying genetic material or therapeutic biomolecules confers exceptional binding or entry specificity, thereby enhancing their delivery efficiency.

EVs are assessed as promising cell-free vaccines for the prevention and control of infectious diseases^[443]^. In 2004, Aline and coworkers reported the effectiveness of EVs derived from *Toxoplasma gondii* antigen-pulsed DC as an innovative cell-free vaccine against toxoplasmosis^[444]^. In addition to their capacity to foster protective immunity against *Toxoplasma* infection, these EVs possess the immunoprophylactic potential against various viral infections. In all, these findings indicated that the EV-based nanomedicine offers a promising strategy for the prevention and blockade of viral infection^[445]^.

#### EVs in bacterial infection

Tuberculosis (TB), caused by *Mycobacterium tuberculosis (M. tuberculosis)*, is a serious disease characterized by symptoms such as blood-tinged mucus, fever, and potentially fatal complications. The emergence of MDR and XDR strains has further exacerbated the challenges in TB treatment^[446]^. To address this issue, Li *et al*. developed a lesion-pathogen dual-targeting strategy for TB treatment, which involved coating mycobacterium-activated macrophage membrane vesicles onto polymeric cores encapsulated with an AIE photothermal agent^[447]^. These engineered vesicles-based nanoparticles can simultaneously target tuberculous granulomas and internalized *M. tuberculosis*. In a TB mouse model, intravenous administration of these nanoparticles effectively eradicated the bacteria, alleviated pathological damage, and reduced excessive inflammation in the lungs, demonstrating superior therapeutic efficacy compared with conventional first-line antibiotics. This membrane vesicle-based dual-targeted photothermal modality represents a promising strategy for TB management.

Infectious wounds are often complicated by persistent MDR bacterial infection and sustained inflammatory responses, which delay the wound-healing process. In addition, excess ROS at the wound site can further impede tissue repair^[448]^. An intelligent dual-layered hydrogel loaded with stem cell-derived vesicles was designed to regulate wound repair^[449]^. Specifically, the inner hydrogel layer responds to bacterial hyaluronidase by releasing ADSC-EVs functionalized with an AIE photosensitizer, which generate ROS upon light irradiation to inhibit bacterial growth. The outer hydrogel layer continuously scavenges excess ROS levels in the wound site, thereby promoting tissue regeneration through the action of ADSC-EVs. This study presents a smart ROS-modulating treatment platform that provides a multifunctional wound dressing for comprehensive repair of infected wounds.

Oral ulcers are refractory superficial lesions of the oral mucosa that are highly susceptible to inflammatory storms and secondary infections. Ge *et al*. constructed a silk fibroin microneedle patch incorporating exosomes derived from LPS-prestimulated BMSCs and zeolitic imidazolate framework-8, localized at the tip and base of the microneedles, respectively^[450]^. Upon coating on the oral superficial lesions, the patch enables sustained release of EVs and Zn^2+^ ions, synergistically enhancing tissue healing and exerting potent antimicrobial effects, thereby accelerating ulcer healing. In another study, EVs derived from Apis mellifera honey demonstrated significant antibacterial activity against oral streptococci, revealing a novel source of EVs enriched with antibacterial peptides^[451]^.

#### EVs in fungal infection

Fungal pathogens can cause many diseases and clinical symptoms by invading the human tissues, triggering inflammatory reactions, inducing tissue damage, and leading to organ dysfunction^[452]^. Recent advances have highlighted the “double-edged sword” role of fungal EVs in mediating intercellular communication and pathogen-host interactions^[453]^. On the one hand, fungal EVs contribute to infection progression and transmission, facilitate fungal-host cell interactions, and deliver virulence factors^[454]^. On the other hand, they can exert protective effects by regulating fungal growth and stimulating adaptive immune responses in the host^[455]^.

Fungal EVs have demonstrated considerable potential for diverse applications. For instance, they can be used to construct drug delivery systems. Moreover, fungal EVs are known to induce protective immune responses against fungal infections, highlighting their promising role in clinical theranotics and disease prevention^[456,457]^. Notably, the surface of fungal EVs contains some known protective antigens, such as those from Mp88 and Gox (galactose oxidase-like) families, providing a strong theoretical support for early prevention and treatment of fungal-related diseases^[458]^. Overall, advances in EV-based nanotechnology are expected to enhance the treatment and prevention of infections by viruses, bacteria, fungi, and other pathogens.

EVs have demonstrated established therapeutic roles in three main areas: as biocompatible drug delivery vectors that improve the bioavailability of therapeutics while mitigating off-target toxicity; as immunomodulators via MSC- or DC-derived EVs that regulate immune cell activity; and as promoters of tissue repair and regeneration through the transfer of bioactive molecules that promote proliferation, extracellular matrix deposition, and neovascularization. Their emerging potential, supported by preclinical studies but still awaiting clinical translation, includes delivering therapeutics across biological barriers for NDs, acting synergistically with photothermal or sonodynamic therapies and immunomodulatory agents, and utilizing microbial EVs as vaccines or drug carriers. However, these applications remain tentative due to unclear mechanisms, limited reproducibility, and a lack of direct clinical evidence.

Notably, although EVs are often thought to act through miRNA delivery, there is ongoing debate about whether the miRNA content in single EVs is sufficient to induce functional changes in recipient cells. This uncertainty underscores key limitations in our current understanding of EV cargo functionality and supports the rationale for engineering strategies - such as overexpression or selective loading of specific miRNAs or other bioactive molecules - to enhance signal intensity and therapeutic reliability.

In different disease contexts, EVs show distinct advantages: tumor-targeting capability in oncology, inherent BBB penetration for neurodegenerative conditions, cardiac repair and anti-inflammatory functions in cardiovascular disease, and the dual targeting-immune activation features of microbial EVs in infections. Nevertheless, they are concomitantly constrained by inherent limitations, such as the potential of tumor-derived EVs to facilitate immune escape, limited brain-targeting efficiency, rapid systemic clearance, and technical challenges in standardizing microbial EV isolation. Broader translational hurdles include insufficient specificity and controllability, difficulties in dose standardization due to EV heterogeneity, and the risk of overgeneralizing preclinical data to clinical settings.

EVs have demonstrated definitive therapeutic value in drug delivery, immunomodulation, and tissue repair, with promising emerging potential in barrier-crossing delivery and combination therapies. In contrast, their actual clinical effectiveness requires systematic validation through well-designed preclinical and clinical studies. Future research should prioritize optimizing EV specificity and controllability, establishing standardized production and quality control systems, and conducting large-scale clinical trials to fully realize their therapeutic potential.

## CLINICAL TRANSLATION CHALLENGES OF EVs

Despite significant progress in the application of EVs for disease diagnosis and therapy over the past decade, several challenges must be addressed before their widespread clinical application can be realized. A major obstacle is the absence of standardized and uniform EV isolation approaches, especially for the large-scale production of therapeutic EVs. In addition, improved characterization technologies and drug-loading methods are also required^[459]^.

Development of standard EV production, isolation, and quality control methods is essential, and efficient isolation and purification of EVs are fundamental prerequisites for their applications in diagnosis and therapy. However, achieving high purity and homogeneity in EV preparations remains highly challenging due to overlapping physicochemical and biochemical characteristics among different EV subpopulations, as well as the inherent heterogeneity in size, composition, and function - even within the same EV subset. Current isolation and analytical methods are far from standardized, which significantly hinders their clinical application. EVs are excellent platforms for the targeted delivery of therapeutic agents, enabling precise treatment strategies. However, limited production yield poses a significant barrier to meeting the demands of a large patient population. Therefore, developing suitable methods and strategies for the scalable generation of EVs is imperative^[318,460]^. Given the intrinsic heterogeneity of EVs and variations in preparation conditions and equipment, manufacturing and purification steps can markedly affect the quality of the final EV products. Thus, establishing a consistent and reproducible production process is essential^[461]^. While several studies have demonstrated the feasibility of small-scale production of EVs under GMP standards, large-scale production remains a considerable challenge^[462]^. Generally, GMP-compliant EV production involves three key stages: upstream cell cultivation, downstream EV purification, and rigorous quality control^[55]^.

Although EVs can be isolated from various biofluids, tissues, and even plants, cell culture systems are often preferred for large-scale production due to their reproducibility, ease of collection, and consistent product stability. Multiple cell types, including stem cells, immune cells, and human embryonic kidney (HEK293) cells, have been utilized for EV production. Among these, HEK293 cells offer distinct advantages, as large-scale culture conditions - such as dissolved oxygen, pH, temperature, and media composition - have been relatively well-established for this cell line. Additionally, the ease of genetic manipulation in HEK293 cells has drawn increasing attention to their use in EV engineering^[463]^. A growing number of studies have employed HEK293 cell-derived EVs for disease therapy^[315]^, highlighting their potential as a standard cell source for scalable EV production and subsequent clinical applications.

In recent years, both flask-based static systems and dynamic culture systems - such as those using shake flasks, spinner flasks, roller bottles, and bioreactors - have been proposed for culturing EV donor cells. While the static systems are straightforward to operate and require less specialized training, making them more commonly used in laboratories, they are unsuitable for large-scale EV production. In contrast, bioreactor systems - including hollow fiber and stirred-tank bioreactors - offer advantages in controllability and automation, making them ideal for large-scale culture to meet clinical and commercial demands^[464,465]^. Notably, 3D culture bioreactor systems overcome the surface area limitations of 2D cultures^[466]^. Moreover, integrating 3D-printed scaffolds with bioreactors has enabled the creation of complex cellular architectures, further enhancing EV production^[467]^. Although such complex bioreactors are still in early development, their modularity of the stiffness and architecture and materials of the 3D-printed scaffold make them highly adaptable to diverse clinical requirements. In line with these advances, a 2025 study reported a scalable 3D bioreactor-based process capable of producing high-yield, high-purity, and cost-effective engineered EVs, demonstrating the practical feasibility of translating such bioprocess designs into clinically oriented, GMP-compatible manufacturing^[468]^.

Quality evaluation is a critical component of routine EV manufacturing and product monitoring. It is essential to establish well-defined CQAs, which should contain the following aspects: (1) Donor cell conditions, including viability and phenotype stability, should be monitored throughout the production process; (2) Physical characteristics (such as particle quantity, size distribution, and surface marker expression) and biological characteristics of purified EVs should be evaluated in accordance with MISEV2023^[469]^; (3) Microbial contamination testing for viruses, endotoxins, and mycoplasma is necessary, as such contaminants may compromise EV efficacy and raise safety concerns^[470]^; (4) Batch-to-batch consistency must be assessed to ensure product uniformity and reproducibility.

In large-scale production of EVs, maintaining their stable activity is a significant challenge^[471]^. The extraction and purification of EVs require precise techniques to ensure their structural and functional integrity. Common separation methods such as ultracentrifugation and SEC, while effective at a small scale, may negatively affect EV integrity and activity when applied at industrial scale. Consequently, TFF is increasingly replacing ultracentrifugation as the preferred option for large-scale separation due to its linear scalability, ease of integration into automated processes, and minimal damage to vesicles^[472,473]^. Additionally, storage conditions, including temperature, pH, and preservation medium, significantly impact EV activity, and must be optimized and justified through stability studies. To ensure that the final EV products meet clinical drug standards, establishing a GMP-compliant Quality Control system is a core prerequisite^[474]^. This requires production in a GMP-compliant environment and a traceable quality management system covering the entire production chain, including strict identification and management of cell sources (Master Cell Bank/Working Cell Bank), characterization of purified EV products according to ISEV guidelines (e.g., particle size distribution, marker protein expression, purity such as particle-to-protein ratio), and comprehensive microbiological safety assessment (sterility, endotoxin, mycoplasma)^[24,475,476]^.

In addition to upstream production and downstream purification, long-term preservation of EVs is another critical bottleneck for clinical translation. Currently, storage at -80 °C is widely regarded as the standard condition to maintain EV integrity and bioactivity; however, the high cost, logistical complexity, and reliance on ultra-low-temperature cold chains significantly limit its feasibility for large-scale commercialization and global distribution. Consequently, there is growing interest in developing lyophilization-based preservation strategies and alternative storage technologies that could enable stable refrigerated or even room-temperature storage of EVs. Recent studies have explored a range of novel cryo- and lyoprotectants - including disaccharides (e.g., trehalose, sucrose), polymers, amino acids, and protein-based excipients - as well as optimized buffer systems and vitrification or spray-drying approaches to minimize vesicle aggregation, membrane damage, and cargo degradation during freezing, drying, and reconstitution. Although most of these technologies remain at the preclinical or early developmental stage, systematic evaluation and standardization of such formulations are needed to establish clinically and commercially viable EV preservation platforms.

Collectively, establishing a standardized operational workflow, encompassing cell culture, EV isolation and purification, long-term storage, and quality control in compliance with GMP principles, is essential before EV-based therapies can advance into clinical translation. However, obtaining large quantities of highly pure and homogeneous therapeutic EVs in a cost-effective and efficient manner remains a major challenge. Furthermore, comprehensive preclinical and clinical assays based on patient demands must be conducted to assess the targeting ability, safety, and efficacy of manufactured EVs. This final step constitutes the most critical pathway for advancing EV-based therapeutics toward clinical translation.

### Efficient loading of therapeutic molecules into EVs

Beyond scalable production, achieving efficient loading of therapeutic molecules into EVs presents another major challenge for EV-related therapy. Currently, two primary strategies are used for loading EVs with cargo: endogenous and exogenous methods^[477]^. Endogenous loading involves incorporating cargo during EV biogenesis through direct transfection, viral infection, or co-incubation of donor cells with drugs. In the co-incubation, the drugs and donor cells were incubated together; thus, the type of donor cells and drugs can significantly influence the loading efficiency. However, the efficiency of co-incubation depends heavily on the cell and drug types, and a major drawback of endogenous approaches is the uncontrollable nature of the cargo loading process, which can result in inconsistent cargo amounts and heterogeneity among EVs. In exogenous methods, therapeutic agents are loaded into prepared EVs mainly using membrane penetration strategies. These approaches allow better control over encapsulation efficiency and cargo loading capacity, making them more suitable for the large-scale manufacturing of therapeutic EVs^[478]^. That said, exogenous loading is generally more applicable to small molecules, and even other nanomaterials^[479]^. Further efforts should focus on developing more efficient and versatile drug loading strategies, such as the combination of above-mentioned approaches, while expanding the range of therapeutic agents compatible with exogenous loading.

### Prospects and pathways of clinical translation of EVs

As pivotal mediators of intercellular communication, EVs exhibit significant potential for applications in disease diagnosis and therapy, owing to their unique biological characteristics and functions. These nanoscale vesicles can carry and deliver a diverse range of biologically active molecules, and offer advantages such as high bioavailability, biological stability, targeting specificity, low toxicity, and low immunogenicity. These attributes make EVs highly promising candidates for clinical translation.

#### EVs for disease diagnosis

Early disease screening, especially for malignant tumors, can effectively improve the 5-year survival rate of patients and even offer a chance for radical cure. Liquid biopsy has a wide range of applications across various diseases. Over the past decade, liquid biopsy techniques, primarily based on circulating tumor cells (CTC) and circulating tumor DNA (ctDNA), have become essential tools for the precise diagnosis of tumors^[480]^. Although EVs (exosomes) hold great potential as a basic component of a liquid biopsy system, their clinical applications still lag behind those of CTC and ctDNA^[481]^.

EV-based biomarkers have been rapidly applied in clinical practice. For example, the first prostate cancer diagnostic test using exosomal RNA has already helped over 50,000 patients in clinical decision-making and has been included in the National Comprehensive Cancer Network Guidelines for Early Detection of Prostate Cancer^[482]^. As emphasized previously, the intrinsic properties of EVs confer unique advantages for early disease screening, disease classification, and prognosis assessment.

Future clinical translation of EVs as medical tools will focus on enhancing diagnostic and therapeutic strategies. Researchers aim to capitalize on the early diagnostic capabilities of EVs, by further exploring the diagnostic information encoded in their specific biomolecular cargo. This will require the development of ultra-sensitive and highly specific detection technologies, enabling not only early disease detection but also precise molecular stratification of pathologies at initial stages, thereby advancing personalized medicine.

For EV-based early disease screening, the preliminary basic research is crucial. Key priorities includes but not limits to: (1) accurate capture of disease-related EVs, such as those derived from tumor cells or other lesion cells; (2) comprehensive understanding of disease mechanisms to allow accurate classification and subtyping; (3) development of signal amplification strategies for EV cargo, given the limited quantity of EVs in bodily fluids during early disease stages, to enable more efficient *in vitro* analysis.

#### EVs for disease treatment

EVs also hold significant translational potential for disease treatment. Among them, MSC-EVs are the most studied, exerting immunomodulatory, anti-inflammatory, anti-fibrotic, and antioxidant effects, as well as promoting angiogenesis, owing to their unique protein and nucleic acid components^[387,483]^. These vesicles have demonstrated considerable clinical value in various human diseases, including neurological, cardiovascular, respiratory, and kidney diseases, as well as metabolic disorders, dermatological conditions, immune regulation, and medical aesthetics^[484-486]^. In particular, the skincare and medical aesthetics industries have been at the forefront of integrating MSC-EVs into commercial products such as creams, essences, masks, and other similar formulations^[487]^. Although MSC-EVs are derived from stem cells and share similar contents to stem cells, they offer improved safety and stability compared to whole stem cells^[48,488,489]^. Therefore, MSC-EVs are expected to advance more rapidly toward clinical translation and application in the near future.

Beyond MSC-EVs, EVs derived from other sources also show high clinical translation potential. For instance, EVs derived from cardiac spheroid-derived cells hold promise for treating Duchenne muscular dystrophy^[490]^, while milk-derived EVs offer broader application prospects due to their superior safety and cost-effectiveness compared with EVs from other sources. Additionally, EVs from DCs have been shown to enhance the body’s ability to clear tumors or infections^[491]^. Despite their promise, these EV-based strategies still require further research and development to effectively advance toward clinical application.

Despite the numerous advantages of EVs as drug delivery vehicles, two major challenges must be overcome before they can be successfully translated into clinical use. First, the large-scale production of EVs remains challenging. Second, although multiple methods exist for drug loading and engineering of EVs, current loading efficiencies are generally low and inconsistent. Compared with natural EVs, engineered EVs show enhanced physicochemical properties and improved therapeutic efficiency. Nevertheless, as discussed previously, clinical translation of EV-based therapies remains hindered by technical challenges, requiring further development before widespread clinical adoption.

LNP-mediated mRNA delivery represents one of the most significant recent advances in drug delivery. Similarly, EVs also offer distinct advantages in delivering small-molecule drugs and nucleic acid therapeutics, particularly mRNA. A direct comparison with EVs highlights a complementary landscape of advantages and challenges for therapeutic applications.

LNPs, although efficacious, can induce acute inflammatory responses and dose-dependent toxicities (e.g., complement activation, hepatic effects)^[492]^. Their cationic or ionizable lipids may cause longer-term accumulation concerns. In contrast, EVs, as natural biological entities, generally exhibit superior biocompatibility and lower inherent toxicity. Their immunogenicity can be tunable; naive EVs may be low-immunogenic or even immuno-tolerant, while engineered EVs can be designed to either avoid immune clearance or specifically modulate immune responses - a dual capability LNPs lack^[493]^.

LNP targeting is primarily passive (e.g., hepatic accumulation via ApoE adsorption) or reliant on the incorporation of complex targeting ligands, which can affect formulation stability. EVs possess intrinsic homing capabilities due to their surface adhesion proteins and glycans^[493,494]^. Furthermore, their natural composition facilitates membrane engineering for enhanced active targeting, enabling more efficient and specific tissue delivery with reduced off-target effects compared to current LNP technologies.

Currently, the major advantage of LNPs lies in their fully synthetic nature, which allows for scalable, GMP-compliant manufacturing with well-defined chemistry, manufacturing, and controls (CMC), resulting in lower and predictable cost of goods sold (COGS). In contrast, EV production faces significant biomanufacturing challenges, including low yields from cell cultures, complex purification requirements, and inherent batch-to-batch variability^[495-497]^. These factors currently result in high COGS, a major hurdle for clinical translation.

In December 2025, regulatory authorities in China classified certain products containing exosomal membrane components, such as exosome-containing hyaluronic acid dressings, as medical devices, thereby offering a reference regulatory route for specific types of EV-based products. In the same year, a Chinese study^[498]^ reported that nebulized administration of human MSC-derived exosomes rapidly improved key pathological and functional parameters in patients with pulmonary fibrosis, further supporting the feasibility and translational potential of inhaled EV-based therapies for fibrotic lung diseases.

## CONCLUSIONS AND FUTURE PERSPECTIVES

As important signal transduction and cellular cargo transportation tools, EVs play a pivotal role in intercellular communications, even across different species, regulating various physiological and pathological processes. Through extensive studies of more than fifty years, EVs represent a quickly evolving research field, and amazing advances have been achieved from basic studies to clinical trials.

Exosomes are an extensively studied EV subgroup. Although the biogenesis of exosomes is relatively clear compared with other EV subpopulations, the regulatory mechanisms mediating selective cargo sorting and secreting of exosomes are not fully elucidated. In addition, standardization in exosome research remains a critical issue. Current isolation and analysis methods are far from standardized. It is still a difficult plan to obtain large-scale exosomes with high purity from different samples, and isolated exosomes are often contaminated by other EV subpopulations or non-vesicular proteins.

EVs have been listed as a key component of a liquid biopsy system for their crucial potential as a non-invasive diagnostic tool. Although clinical trials are ongoing to evaluate their diagnostic value, many questions remain unsolved. Among these, the heterogeneity of EVs is a major obstacle for developing standardized EV-based diagnostic tools for human diseases. More accurate methods are needed to isolate and identify distinct EV subpopulations with different cellular origins and functions.

The rapid advancement of artificial intelligence (AI) technologies, especially deep learning, and machine learning, has boosted the application of EVs in disease diagnosis and therapy. Based on the AI-driven multi-omics analyses and machine learning algorithms, EV-based biomarker discovery, disease prediction, precision treatment, and therapeutic monitoring have increasingly been reported^[499-501]^. Moreover, integration of the AI with EV engineering may provide possibility to circumvent the translational barriers, including loading efficiency, targeted delivery, *in vivo* stability, and pharmacokinetics, immunogenicity, and safety^[502]^. Since the performance of the deep learning and machine learning-based AI techniques is contingent on the data quality, future regulatory frameworks and standardized procedures should be developed to ensure the credibility and reliability of collected data. With the continued advance in AI technology, EV-based disease diagnosis and therapies are expected to expand significantly, enabling repeatable and replicable models and paving the way for personalized therapy and precision medicine.

As “natural nanoparticles”, EVs display high biocompatibility, circulating stability, the ability to cross biological barriers, and low immunogenicity, highlighting their potential as both therapeutic modalities and drug carriers. Although underlying mechanisms have not been well elucidated, multiple clinical trials have shown substantial therapeutic potential of specific EVs such as MSC-EVs in certain human diseases. A variety of engineered EVs have been developed to deliver therapeutic drugs to target tissues and cells. However, no EV-based drug delivery system has been approved for clinical use, partly due to lack of standard purification methods and extensive safety evaluation. Preclinical investigations have confirmed that surface modification of EVs could promote targeting abilities of engineered EVs to special cell types. However, challenges remain in increasing their circulating stability and reducing their accumulation in non-target tissues, particularly in the liver, during systemic administration. In addition to mammalian cells, plants, milk, and even microbial pathogens are also potential sources of EVs for specific aim of drug delivery or EV-based vaccines, providing additional avenues for EV-based therapeutic applications.

Due to limited size and loading efficiency of EVs, it remains challenging to load sufficient quantities of drugs or therapeutic molecules, especially those with large molecular weight. In addition, the complex surface structure of EVs and limited size are potential obstacles for efficient surface labeling. In addition to improving cargo loading and labeling techniques, modifying the surface structure of EVs may represent an alternative strategy. For example, trypsin treatment can cleave extracellular domains of membrane proteins on the exosome surface, reducing their potential hindrance for cargo loading and surface modifications^[503]^. Moreover, recent studies confirmed the feasibility of oral administration of EVs for disease therapy. These new advances are expected to accelerate the clinical translation of EV-based therapies in the future.

## DECLARATIONS

### Copyright

© The Author(s) 2026.
